# Efficacy and Safety of SPN-812 (Extended-Release Viloxazine) in Children and Adolescents with Attention-Deficit/Hyperactivity Disorder: A Systematic Review and Meta-Analysis

**DOI:** 10.3390/brainsci13121627

**Published:** 2023-11-24

**Authors:** Xin Tan, Yuejuan Xu, Shixin Wang, Jiaxuan Li, Chunxia Hu, Zhouqing Chen, Qingzhang Cheng, Zhong Wang

**Affiliations:** 1 Department of Neurology, The Affiliated Suzhou Hospital of Nanjing Medical University, Suzhou Municipal Hospital, Suzhou 215000, China; xintan862968596@163.com; 2 Department of Respiratory Medicine, Children’s Hospital of Wujiang District, Children’s Hospital of Soochow University, Suzhou 215025, China; xuyuejuan96@163.com (Y.X.); huchunxia780701@163.com (C.H.); 3 Department of Neurosurgery& Brain and Nerve Research Laboratory, The First Affiliated Hospital of Soochow University, Suzhou 215006, China; wsx9036@163.com (S.W.); lijiaxuan1622@163.com (J.L.); zqchen6@163.com (Z.C.); 4 Department of Neurosurgery, The First Affiliated Hospital of Soochow University, Suzhou 215006, China

**Keywords:** SPN-812, viloxazine, pediatric patients, attention-deficit/hyperactivity disorder, systematic review

## Abstract

Background: SPN-812 has been approved for attention-deficit/hyperactivity disorder (ADHD) treatment in children and adolescents. Objective: We aimed to analyze the efficacy and safety of different doses of SPN-812 for ADHD pediatric patients of different ages, verify its clinical efficacy, and evaluate its safety. Methods: Up until 30 August 2023, randomized controlled trials (RCTs) were searched in EMBASE, MEDLINE, the Cochrane Library, and clinicaltrials.gov to evaluate different doses of SPN-812 and a placebo. Results: We pooled 1619 patients from five RCTs with a duration of 6–8 weeks. Patients (6–17 years old) in SPN-812 (100, 200, and 400 mg/d) groups were superior to the control group in all efficacy outcomes with lower attention-deficit/hyperactivity disorder rating scale-5 (ADHD-RS-5), Conners 3-parent short form composite T score (Conners 3-PS), Weiss functional impairment rating scale-parent (WFIRS-P), and increased clinical global impression-improvement (CGI-I) score (both *p* < 0.05). At the same time, only SPN-812 300 mg/d did not show a significantly high risk of the adverse events (AEs) such as somnolence and decreased appetite (*p* = 0.09). There was no significant difference between placebo and SPN-812 groups (100, 200, and 400 mg/d) in serious adverse events (SAEs) such as syncope. The subgroup analyses showed that, both in children and adolescents subgroups, SPN-812 showed better efficacy than the placebo. The two age subgroups showed a significantly higher risk of AEs and an insignificant risk of SAEs than the placebo. Conclusion: At present, SPN-812 (100, 200, and 400 mg/d) is superior to the corresponding control in efficacy measures. However, the safety problem cannot be ignored.

## 1. Introduction

Attention-deficit/hyperactivity disorder (ADHD) is a common and impairing neuropsychiatric disorder affecting children and adolescents, and it is characterized by age-inappropriate and impairing levels of inattention, impulsivity or hyperactivity, or a combination [1]. It is estimated that 5% of pediatric patients aged <18 years and 2.5% of adults are affected by this disorder worldwide [2]. In clinical practice, ADHD patients often have considerable heterogeneity in symptom trajectories, clinical presentation, and treatment response, making clinical diagnosis and management challenging [3]. In addition to the core dysfunction of ADHD, individuals are at increased risk of psychiatric comorbidities including drug abuse, impaired occupational, academic, and health maintenance [4,5]. The economic burden of ADHD among children and adolescents is substantial, driven by expenses for care, education, and healthcare. According to a recent research report from Florida International University, the estimated annual cost of illness for ADHD in children and adolescents is approximately US 124.5 billion, underscoring the necessity for intervention [6].

With the continuous exploration of ADHD in recent years, the understanding of the disease is continuously updated. Currently, medications, nutrition management, behavior therapy, exercise, and social skills training, have been applied in practice for interventions of ADHD [3,7]. For instance, recent research found that neurofeedback plus vitamin D supplementation works better together than therapy alone to treat children with ADHD and can decrease the duration of treatment [8]. To date, there are a variety of pharmacological treatment options, such as stimulant classes (amphetamine and methylphenidate) which are recommended as the first line of treatment in children and adolescents with ADHD [1,9], and some non-stimulants, which act as receptor agonists for epinephrine and norepinephrine systems [10]. The potential advantages of stimulants are that they have rapid onset of treatment effects and are able to greatly reduce the core symptoms of ADHD [11]. At the same time, there are short-acting and long-acting formulations for stimulants, as well as different forms such as liquids, chewing pills, and transdermal patches. Whereas, their disadvantages are also clear. For example, the duration of daily efficacy is limited. When the effect disappears in the afternoon/evening, some symptoms may rebound [12]. Approximately 25 percent of children with ADHD are unresponsive to stimulants. At the same time, amphetamine and methylphenidate are sympathomimetic drugs, which can affect mood, growth, appetite, blood pressure, pulse and sleep patterns and participate in increasing aggression, anxiety, and hallucinations [13]. Finally, patients discontinue treatment due to the intolerable side effects of these drugs despite persistent symptoms [14]. On the other hand, for non-stimulants, the advantages are that they can have an “Around-the-clock” effect and may enhance the treatment of stimulants [15]. Furthermore, for patients with comorbid disruptive behavior disorders, tic/Tourette’s disorder, and substance use disorders, non-stimulants seem to be a better choice [16]. But their effect size is relatively smaller than that of stimulants, and they have a slower onset of action [12,17]. In short, ADHD treatments still have significant limitations. Therefore, for patients who are not suitable for current stimulant and/or non-stimulant prescriptions, effective and tolerable alternative drugs, especially non-stimulants, are needed.

SPN-812 (extended-release viloxazine), is known as an effective and well-tolerable agent for ADHD treatment. As a bicyclic norepinephrine reuptake inhibitor with selective serotonergic activity, SPN-812 is a novel, non-stimulant medication currently for the treatment of ADHD in children and adolescents [18]. (a) Mechanism of action: the mechanism of SPN-812 is unique, as a serotonin-norepinephrine modulating agent (SNMA), compared with other ADHD and depression pharmacotherapies. As a selective 5-HT_2B_ receptor antagonist and 5-HT2C receptor agonist, SPN-812 modulates serotonergic activity and moderately inhibits norepinephrine transporter (NET), thus blocking the reuptake of norepinephrine [19]. (b) US Food and Drug Administration (FDA) Approval: in 2021, SPN-812 has been approved for treatment of ADHD in children aged 6–17 [20].

The efficacy and safety of SPN-812 have been adequately demonstrated in a number of randomized controlled trials (RCTs) over the past decade. In the last year, a meta-analysis showed that viloxazine was significantly superior to placebo in patients with ADHD (6–17 years of age), which compared only two-dose groups of 200 mg/d and 400 mg/d in subgroup analysis [21]. Similarly, a narrative review was conducted to confirm that SPN-812 was an effective, well-tolerated alternative for some children with ADHD [22]. Moreover, less systematic comparisons of different SPN-812 doses exist, since once-daily doses of 100 mg, 200 mg, 300 mg, 400 mg, and 600 mg could induce clinical improvement in ADHD symptoms in pediatric patients compared with the placebo group. Whether lower doses of SPN-812 can preserve its effectiveness while reducing adverse events and patient costs remains to be revealed. Which is the best SPN-812 dose for pediatric ADHD patients? Thus, in order to evaluate the differences in efficacy and safety of various dosages of SPN-812, we aggregated data from prior RCTs [18,23,24,25,26] and carried out a meta-analysis. At the same time, we tried to answer the question mentioned above.

## 2. Methods

### 2.1. Study Protocol

Before conducting the investigation, we designed a research protocol following the Cochrane Collaboration framework, which provided a standard to follow for any researcher seeking to write a methodologically sound and transparent systematic literature review [27]. The protocol for this systematic review was registered on INPLASY with the number INPLASY202390059.

### 2.2. Eligibility Criteria

We defined the following inclusion criteria: (1) study design: RCT; (2) language limitation: available only in English; (3) participants: children (6–11 years old) and adolescents (12–17 years old) diagnosed with ADHD; (4) intervention: different doses of SPN-812 and placebo; (5) outcomes: efficacy outcomes including attention-deficit/hyperactivity disorder rating scale-5 (ADHD-RS-5), the number of patients with increased clinical global impression-improvement (CGI-I) score, Conners 3-parent short form composite T score (Conners 3-PS), the Weiss functional impairment rating scale-parent (WFIRS-P), adverse events (AEs), and serious adverse events (SAEs) were included in the assessment of safety outcomes. The included RCTs were not required to provide all of the above-mentioned outcomes.

We established the following exclusion criteria: (1) study design: case reviews, case reports, retrospective studies, and cohort studies; (2) control: active control (i.e., known effective treatments compared to experimental treatment rather than a placebo).

### 2.3. Search Process

To find pertinent papers published up to 30 August 2023, two independent researchers (XT and YJX) conducted a thorough search of Clinicaltrials.gov and three major databases: MEDLINE, EMBASE, and the Cochrane Library, and attempted to identify unpublished or gray literature. The following search strategies were used: (SPN-812[Title/Abstract]) AND (attention-deficit/hyperactivity disorder [Title/Abstract]) for MEDLINE; ‘SPN-812’/exp AND ‘attention-deficit/hyperactivity disorder’/exp for EMBASE; “SPN-812” in Title Abstract Keyword AND “attention-deficit/hyperactivity disorder” in Title Abstract Keyword for Cochrane Library; “SPN-812 | attention-deficit/hyperactivity disorder” for Clinicaltrials.gov. In addition, we performed an independent manual screening of reference lists for RCTs and related systematic analysis to avoid omissions in our search. The search strategy was presented (Table S2).

### 2.4. Study Selection and Data Collection

Following the eligibility criteria mentioned above, there were three reviewers involved in the selection process who were trained. Two reviewers (XT and YJX) independently assessed each trial record from the electronic databases, as well as the reference lists of related RCTs, systematic reviews, and meta-analyses. The research literature providing only abstracts and duplicate articles were excluded, because they could not provide complete data for further analysis. When there was a disagreement between the two reviewers, it could be resolved through discussion. A third reviewer (SXW), although not involved in the data collection process, would make a final decision on the controversial data when a disagreement occurs between the two reviewers and it cannot be resolved through discussion. All the data from the included RCTs were carefully selected, evaluated, and then summarized as follows: for each trail, basic data and outcome events were presented (Table 1), study design, inclusion and exclusion criteria, and all efficacy and safety measures were shown in the online Supplementary Materials (Table S1). Microsoft Excel software 2021 was used for data collection and extraction.

### 2.5. Analysis of Bias Risk

The Review Manager 5.3 software was applied to evaluate the risk of bias for every study. Bias, also known as a systematic error, refers to the tendency of a research result system to deviate from the true value. The unbiased research results have good intrinsic authenticity, which is significant for meta-analysis. The uniform criteria for evaluating bias risk of the Cochrane Collaboration included detection bias, attrition bias, selection bias, performance bias, reporting bias, and other potential biases. The third reviewer independently assessed each bias criteria and assigned it to one of three categories: “low”, “high”, or “unclear”. Here, “low” means that the existence of bias cannot greatly affect the research results; “high” means that the existence of bias can largely weaken the credibility of the study; “unclear” means that the existence of bias can weaken the credibility of the research. For example, for selection bias, if the RCT used a random number table for randomization allocation, the bias category is “low”. If the RCT used the alternate allocation method for randomization, the bias category is “high”. If the information in the text is not clear, the bias category is “unclear”.

### 2.6. Summary Measures and Synthesis of Results

Data were assessed by Review Manager 5.3 software. The risk ratio (relative risk [RR]; 95% confidence interval [CI]) was applied to calculate the dichotomous variable with a random effect model. For the ADHD-RS-5 score, Conners 3-PS score, WFIRS-P score, and other continuity indicators, the mean difference (MD) was used for statistics. I^2^ statistic was used to evaluate heterogeneity as follows: “low heterogeneity” corresponded to I^2^ < 30%; “moderate heterogeneity” was represented by I^2^ between 30% and 50%; I^2^ > 50% indicated “substantial heterogeneity”. The stability of the consolidate results is evaluated using sensitivity analysis. Additionally, by grouping patients of different ages at baseline, subgroup analyses were conducted. Two-tailed tests were used for all of the analyses, and a *p* value of 0.05 or lower was regarded as statistically significant.

## 3. Results

**Figure 1:**
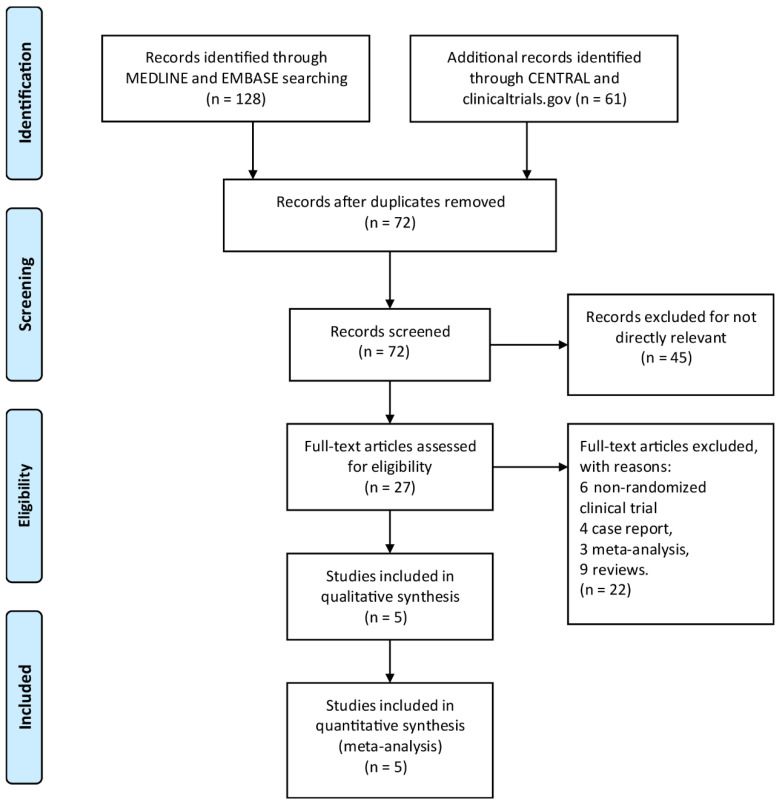
The study search, selection, and inclusion process.

By searching through EMBASE, Cochrane Library, MEDLINE, and Clinicaltrials.gov, a total of 189 titles and abstracts were returned. Moreover, a total of 162 articles were excluded for irrelevance to the research topic and duplication by screening article titles and abstracts, and 27 full-text articles were assessed as qualified. Of the aforementioned 27 articles, 22 articles were excluded due to inappropriate publication types, including 4 case reports, 6 non-randomized clinical trials, 3 meta-analyses, and 9 reviews. The detailed article selection process is summarized in the flowchart (Figure 1). Finally, we include a total of five RCTs [18,23,24,25,26] with 1619 patients enrolled. Table 1 is a summary of the main features of the five studies.

### 3.1. Efficacy Outcomes Analysis

The efficacy outcomes included the ADHD-RS-5, the number of patients with increased CGI-I score, Conners 3-PS, and the WFIRS-P. Patients in SPN-812 100 mg/d, 200 mg/d, and 400 mg/d group had significantly lower ADHD-RS-5, Conners 3-PS, and WFIRS-P score than those in the control group (MD < 0, 95% CI excludes 0). There were statistically significant differences in terms of the number of patients with elevated CGI-I scores between the control group and the groups receiving the three different dosages of SPN-812 (100 mg/d: RR = 1.51 [1.12, 2.04], *p* = 0.007; 200 mg/d: RR = 1.57 [1.30, 1.88], *p* < 0.00001; 400 mg/d: RR = 1.58 [1.30, 1.91], *p* < 0.00001). However, there was no statistically significant difference between the SPN-812 600 mg/d group and the placebo group. Table 2 and Figures S1–S4 show the detailed findings of the clinical outcome analysis.

### 3.2. Safety Outcomes Analysis

The assessment of adverse events and serious adverse events following administration was included in the safety outcome. We integrated data from the five studies into our analysis and discovered that only SPN-812 at 300 mg/day did not have a substantially high risk of adverse events (RR = 1.50 [0.93, 2.41], *p* = 0.09, Table 2 and Figure S5). We were unable to collect data on the performance of SPN-812 300 and 600 mg/d in SAEs. For the collected data, there was no significant difference between placebo and any dosage of SPN-812 in SAEs (for SPN-812 100 mg/d: *p* = 0.29; for SPN-812 200 mg/d: *p* = 0.16; for SPN-812 400 mg/d: *p* = 0.14, Table 2 and Figure S6).

### 3.3. Subgroup Analyses

To assess the influence of different age groups (children and adolescents), we implemented subgroup analyses at the baseline. The results indicated that, although SPN-812 showed a significantly better efficacy than placebo both in children and adolescents, the effect seemed more pronounced in children. In the subgroup analysis of safety outcomes, we did not find much difference between the two age subgroups given that they both showed a significantly higher risk of AEs and an insignificant risk of SAEs than the placebo group. The detailed results of subgroup analyses were shown in Table 3 and Figures S7–S12.

### 3.4. Risk of Bias in Enrolled Studies

**Figure 2:**
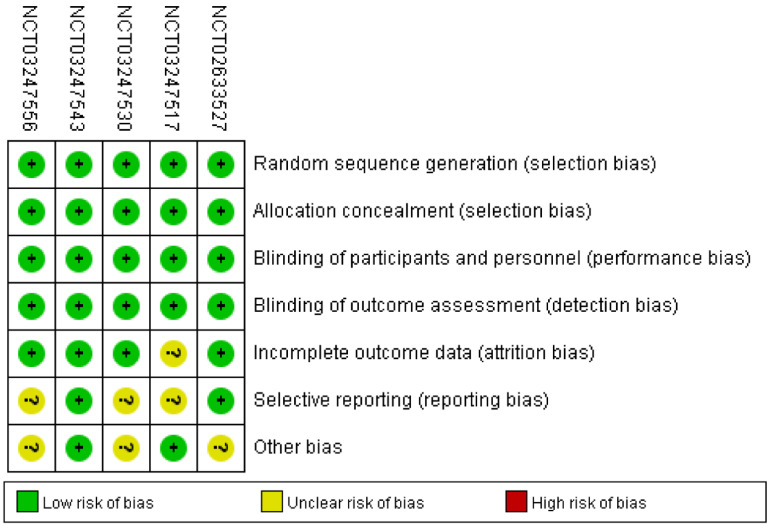
Risk of bias: A summary table for each risk of bias item for each study.

Figure 2 showed comprehensive details on the risk bias for all included studies. In terms of allocation concealment and random sequence creation, all five clinical trials displayed low bias risk. The risk of bias was also low across all five trials for the blinding of participants, staff, and outcome assessment. The risk of bias was uncertain due to the insufficient outcome data in only one trial. With respect to selective reporting, the risk of bias in the three trials was not clear. Along with these items, unclear bias risks could also be found in three RCTs.

## 4. Discussion

Nowadays, as a novel non-stimulant medication, SPN-812 (extended-release viloxazine) has been approved by the FDA under the trade name “Qelbree” for the treatment of ADHD in children and adolescents (ages 6–17 years) [20]. The present analysis was based on five double-blind trials that included children and adolescents (6–17 years old) diagnosed with ADHD who were randomly assigned to different doses of SPN-812 and placebo, and tried to evaluate its efficacy and safety through the following indicators.

According to the Diagnostic and Statistical Manual of Mental Disorders (fifth edition), the ADHD-RS-5 is a validated scale of 18 items which can be further subdivided into subscales: inattention (9 items) and hyperactivity/impulsivity (9 items). According to the findings of our pairwise meta-analysis, patients in SPN-812 (100 mg/d, 200 mg/d, and 400 mg/d) groups had significantly lower ADHD-RS-5 than those in the control group, indicating that the severity of clinical symptoms could be reduced and a significant number of patients in this study experienced a clinical improvement across the spectrum of core ADHD symptoms after the SPN-812 treatment. Moreover, the CGI-I is a single-item rating reflecting the clinician’s assessment of the deterioration or improvement of the disease relative to baseline, which was based on the interview with a parent or subject. It is rated on a 7-point scale where 1 = very much improved, 2 = much improved, 3 = minimally improved, 4 = no change, 5 = minimally worse, 6 = much worse, and 7 = very much worse [28]. Previous study indicated that a great symptomatic improvement on the ADHD-RS-5 (50–60% reduction) is in concordance with CGI-I score of 2 (much improved) [29]. And correspondingly, in our study, significant differences were observed in the number of patients with increased CGI-I score between control group and three different doses of SPN-812 group (100 mg/d, 200 mg/d, and 400 mg/d), which was consistent with ADHD-RS-5 results and indicated a clinically meaningful improvement of SPN-812 treatment. These results are consistent with the published Phase III pivotal trial assessing the efficacy and safety of SPN-812 in adults with ADHD. Similarly, the CGI-I scores were greatly improved relative to placebo after 6 weeks of treatment with SPN-812, suggesting a considerable agreement between adult and pediatric studies [28].

Conners 3-PS covers six content scales to assess ADHD-related behavior: learning problems, inattention, hyperactivity, executive functioning, peer relations, and aggression. As functional impairment is central to ADHD, the WFIRS-P, a 50-item scale, is used to assess functional impairment on six clinically relevant domains including: self-concept, family, life skills, school, social activities, and risky activities [30]. For instance, it was applied to assess the severity of the impairment of subjects’ ability to accomplish daily tasks and interactions due to their emotional and behavior problems [31]. The lower the score, the greater the improvement of clinical symptoms. In our study, patients treated with SPN-812 (100 mg/d, 200 mg/d, and 400 mg/d) had significantly lower Conners 3-PS and WFIRS-P scores than those in the control group, which presented that SPN-812 played a positive role in the improvement of behavior and functional disorders for children and adolescents with ADHD. However, patients in SPN-812 600 mg/d group did not show significant differences in all four efficacy outcomes when compared with the placebo. Since there was only one RCT set up as a 600 mg/d group; therefore, this dose group cannot be arbitrarily regarded as a poor effect. On the other hand, actually, the lack of an apparent dose–response relationship for psychotropic medications is common in clinical trials for numerous reasons such as non-linear effects at the site of action [23]. For instance, recently, when researching the dose–response relationship of selective serotonin and norepinephrine reuptake inhibitors (SNRIs) in the treatment of depression, researchers discovered that increasing the dose did not provide a clinically and statistically meaningful change [32], maybe this is because the concentration of drug administered could not be completely equivalent to the concentration in blood.

The subgroup analyses showed that, both in children and adolescent subgroups, SPN-812 showed better efficacy than the placebo. Regarding safety, our data suggested that only SPN-812 300 mg/d did not show a significantly high risk of the AEs. However, the sample size of SPN-812 at the dose of 300 mg/d was not large. Both the low dose of 100 mg/d and the relatively high dose of 600 mg/d showed a higher risk of AEs compared with the control group. In general, a larger dose is often accompanied by more adverse reactions, but since there is only one RCT data in the 600 mg/d group of this study, further studies need to be included to obtain more accurate analysis. In addition, the minimum dose for all studies is 100 mg/d, which also indicates the risk of AEs. This makes us wonder if there is a lower dose that better balances efficacy and safety. Meanwhile, in the subgroup analysis of safety outcomes, both children and adolescent groups showed a significantly higher risk of AEs than the placebo group. At present, it is believed that the most common adverse reactions for SPN-812 are somnolence, decreased appetite, fatigue, vomiting, nausea, insomnia, and irritability [20]. Due to potential somnolence and fatigue, patients need to pay more attention to safety when performing operations that require high concentration. Other warnings include possible activation of mania or hypomania [13]. Whereas, based on previous studies, SPN-812 was well tolerated generally, with most AEs being mild or moderate in severity [28,33]. It is consistent with our findings, for the collected data, there was no significant difference between the placebo and any dosage of SPN-812 (100 mg/d, 200 mg/d, and 400 mg/d) in SAEs. In the subgroup analysis, there was no significant difference between children and adolescents given that they both showed an insignificant risk of SAEs than the placebo group. Currently, the recommended starting dose for patients aged 6–11 years is 100 mg once daily and according to the patient’s actual medication tolerability and response it can be titrated in increments of 100 mg weekly to the maximum recommended dosage of 400 mg once daily. Similarly, for patients aged 12 to 17 years, the starting dosage can be doubled (200 mg once daily). It can be titrated in increments of 200 mg weekly after the first week of administration to the maximum recommended dosage of 400 mg once daily [20]. Combined with our analysis, we believe that more and large-scale high-quality studies are needed. Trials of a lower dose are still of great clinical significance to determine the best dose for both efficacy and safety because the lack of an apparent dose–response relationship is common for psychopharmacotherapy. Currently, guidelines for ADHD treatment in children and adolescents recommended stimulant or non-stimulant medication. However, the stimulant therapy, particularly the immediate-release formulations, had a risk of diversion and misuse [34,35] and was recognized to be associated with episodes of psychosis [36]. At the same time, it is believed that stimulants provide variable efficacy throughout the day [37]. Relevant research data showed that, with the prescription use of the stimulants, approximately 20% to 40% of patients with ADHD failed to achieve great treatment response or symptomatic remission [38,39,40]. It was worth noting that there were no pharmacokinetic interactions between SPN-812 and ADHD stimulant treatments such as methylphenidate and lisdexamfetamine [33,41]. Meanwhile, the pharmacokinetics of SPN-812 were not affected by strong cytochrome P450 (CYP) 2D6 inhibitors such as paroxetine [42]. Therefore, SPN-812 may be a promising complementary or alternative therapy for patients who do not respond well to stimulant therapy. In addition, SPN-812 has other advantages. In contrast to traditional non-stimulant ADHD medication, which cannot be sprinkled over the food, SPN-812 can be swallowed whole or opened and the entire contents sprinkled onto the food, which provides an effective solution for adolescents with ADHD who may be accompanied by autism or other developmental disorders, making it difficult to swallow complete tablets/capsules [43]. Above all, SPN-812 can be an important supplementary treatment for children and adolescents with ADHD.

Several limitations of the present meta-analysis should not be ignored. Firstly, the analysis was performed based on limited data, only five published RCTs were pooled to test the efficacy and safety of SPN-812. Due to the fact that only one RCT (NCT03247556) with a dose of 600 mg/d has been included, the clinical efficacy of this dose needs to be confirmed through more high-quality, larger sample studies in the future. Furthermore, the follow-up duration is relatively short, a longer trial duration may be required to evaluate the long-term efficacy and safety of SPN-812. Secondly, most of the included studies were conducted by the same group of researchers; therefore, there may be some bias. Thirdly, the majority of subjects were Caucasians. The lack of experimental data from different ethnic groups may affect the applicability of the results worldwide. More diverse data are needed to ensure the generalizability of the findings to different ethnic groups.

## 5. Conclusions

In short, our study presented that SPN-812 (100 mg/d, 200 mg/d, and 400 mg/d) is superior to the corresponding control in efficacy measures. However, the safety problem needs to be paid more attention, and it is still of great clinical importance to further explore the efficacy and safety of the low dose of this drug. Additional large-scale and high-quality studies including diverse ethnic groups are still needed to further explore the safety of this therapy. Finally, early diagnosis and early treatments are recognized to be vital for children and adolescents with ADHD.

## Figures and Tables

**Table 1:** Characteristics of the included studies and outcome events.

Study	Centers	Treatment Group (No. of Participants)	Male (%)	Mean Age ± SD (Year)	Study Period (Weeks)	Outcome Events
Nasser A et al., 2021 [26] (NCT03247556)	27	SPN-812 400 mg (99) vs. SPN-812 600 mg (97) vs. Placebo (96)	SPN-812 400 mg: 66.7 SPN-812 600 mg: 73.2 Placebo: 63.5	SPN-812 400 mg: 14.0 ± 1.74 SPN-812 600 mg: 13.7 ± 1.52 Placebo: 13.8 ± 1.53	7	a, b, c, d, e, f
Johnson et al., 2019 [18] (NCT02633527)	32	SPN-812 100 mg (45) vs. SPN-812 200 mg (46) vs. SPN-812 300 mg (47) vs. SPN-812 400 mg (44) vs. Placebo (24)	SPN-812 100 mg: 60.0 SPN-812 200 mg: 71.7 SPN-812 300 mg: 76.6 SPN-812 400 mg: 70.5 Placebo: 45.8	SPN-812 100 mg: 8.2 ± 1.36 SPN-812 200 mg: 9.0 ± 1.36 SPN-812 300 mg: 9.0 ± 1.36 SPN-812 400 mg: 9.0 ± 1.36 Placebo: 8.9 ± 1.28	8	a, e
Nasser A et al., 2021 [23] (NCT03247517)	33	SPN-812 200 mg (94) vs. SPN-812 400 mg (103) vs. Placebo (104)	SPN-812 200 mg: 70.2 SPN-812 400 mg: 65.0 Placebo: 55.8	SPN-812 200 mg: 13.9 ± 1.48 SPN-812 400 mg: 14.0 ± 1.59 Placebo: 13.8 ± 1.60	6	a, b, c, d, e, f
Nasser A et al., 2020 [24] (NCT03247530)	34	SPN-812 100 mg (147) vs. SPN-812 200 mg (158) vs. Placebo (155)	SPN-812 100 mg: 63.9 SPN-812 200 mg: 62.7 Placebo: 62.6	SPN-812 100 mg: 8.5 ± 1.7 SPN-812 200 mg: 8.5 ± 1.7 Placebo: 8.5 ± 1.7	6	a, b, c, d, e, f
Nasser A et al., 2021 [25] (NCT03247543)	28	SPN-812 200 mg (107) vs. SPN-812 400 mg (97) vs. Placebo (97)	SPN-812 200 mg: 69.2 SPN-812 400 mg: 60.8 Placebo: 62.9	SPN-812 200 mg: 8.5 ± 1.7 SPN-812 400 mg: 8.4 ± 1.7 Placebo: 8.5 ± 1.7	8	a, b, c, d, e, f

SPN-812: extended-release viloxazine; a: attention-deficit/hyperactivity disorder rating scale-5 (ADHD-RS-5); b: clinical global impression-improvement (CGI-I); c: Conners 3-parent short form composite T score (Conners 3-PS); d: Weiss functional impairment rating scale-parent (WFIRS-P); e: adverse events (AE); f: serious adverse events (SAE).

**Table 2:** Effect sizes from the meta-analysis of efficacy and safety outcomes as well as from all trials using the random effect models.

Outcomes	No. of Trials Contributing to the Meta-Analysis	No. of Participants Contributing to the Meta-Analysis	MD (95% CI)/RR [95% CI]	*p* Value	I^2^ (%)
1. ADHD-RS-5					
SPN-812 100 mg	2	371	−5.79 (−8.68, −2.90)	<0.0001	0
SPN-812 200 mg	4	785	−6.10 (−8.10, −4.09)	<0.00001	0
SPN-812 300 mg	1	71	−8.10 (−14.93, −1.27)	0.02	N/A
SPN-812 400 mg	4	664	−5.61 (−7.76, −3.46)	<0.00001	0
SPN-812 600 mg	1	193	−3.50 (−7.34, 0.34)	0.07	N/A
2. Number of patients with increased CGI-I score					
SPN-812 100 mg	1	302	1.51 [1.12, 2.04]	0.007	N/A
SPN-812 200 mg	3	715	1.57 [1.30, 1.88]	<0.00001	0
SPN-812 400 mg	3	596	1.58 [1.30, 1.91]	<0.00001	0
SPN-812 600 mg	1	193	1.38 [0.98, 1.95]	0.07	N/A
3. Conners 3-PS					
SPN-812 100 mg	1	302	−4.30(−6.58, −2.02)	0.0002	N/A
SPN-812 200 mg	3	715	−3.13(−5.27, −1.00)	0.004	49
SPN-812 400 mg	3	596	−2.37(−3.93, −0.80)	0.003	0
SPN-812 600 mg	1	193	−1.30(−3.75, 1.15)	0.30	N/A
4. WFIRS-P					
SPN-812 100 mg	1	302	−0.14 (−0.23, −0.05)	0.002	N/A
SPN-812 200 mg	3	715	−0.13 (−0.19, −0.07)	<0.0001	0
SPN-812 400 mg	3	596	−0.10 (−0.16, −0.04)	0.0008	0
SPN-812 600 mg	1	193	0.00 (−0.08, 0.08)	1.00	N/A
5. AEs					
SPN-812 100 mg	2	385	1.60 [1.25, 2.05]	0.0002	0
SPN-812 200 mg	4	805	1.31 [1.11, 1.56]	0.002	8
SPN-812 300 mg	1	72	1.50 [0.93, 2.41]	0.09	N/A
SPN-812 400 mg	4	682	1.61 [1.21, 2.13]	0.001	70
SPN-812 600 mg	1	196	1.81 [1.39, 2.36]	<0.00001	N/A
6. SAEs					
SPN-812 100 mg	1	313	5.16 [0.25, 106.64]	0.29	N/A
SPN-812 200 mg	3	733	3.61 [0.59, 22.00]	0.16	0
SPN-812 400 mg	2	400	5.00 [0.59, 42.40]	0.14	0

MD: mean difference; RR: relative risk; CI: confidence interval; ADHD-RS-5: attention-deficit/hyperactivity disorder rating scale-5; CGI-I: clinical global impression-improvement; Conners 3-PS: Conners 3-parent short form; WFIRS-P: Weiss functional impairment rating scale-parent; AEs: adverse events; SAEs: serious adverse events.

**Table 3:** Subgroup analysis of efficacy and safety outcomes.

	Children (6–11 Years)		Adolescents (12–17 Years)	
	MD (95% CI)/RR [95% CI]	*p* Value	MD (95% CI)/RR [95% CI]	*p* Value
Efficacy outcomes				
ADHD-RS-5	−6.33 (−8.37, −4.30)	<0.00001	−4.60 (−6.94, −2.25)	0.0001
Number of patients with increased CGI-I score	1.50 [1.22, 1.83]	<0.0001	1.59 [1.28, 1.98]	<0.0001
Conners 3-PS	−3.92 (−5.43, −2.40)	<0.00001	−1.72 (−3.35, −0.09)	0.04
WFIRS-P	−0.14 (−0.20, −0.08)	<0.00001	−0.07 (−0.13, −0.01)	0.02
Safety outcomes				
AEs	1.40 [1.15, 1.71]	0.0008	1.52 [1.24, 1.85]	<0.0001
SAEs	3.52 [0.44, 28.47]	0.24	5.05 [0.59, 42.82]	0.14

MD: mean difference; RR: relative risk; CI: confidence interval; ADHD-RS-5: attention-deficit/hyperactivity disorder rating scale-5; CGI-I: clinical global impression-improvement; Conners 3-PS: Conners 3-parent short form; WFIRS-P: Weiss functional impairment rating scale-parent; AEs: adverse events; SAEs: serious adverse events.

## Data Availability

This published article and its supplemental files contain all of the data created or analyzed throughout this investigation.

## Supplementary Materials

The following supporting information can be downloaded at: https://www.mdpi.com/article/10.3390/brainsci13121627/s1, Figure S1: Meta-analysis of efficacy: ADHD-RS-5; Figure S2: Meta-analysis of efficacy: Number of patients with increased CGI-I score; Figure S3: Meta-analysis of efficacy: Conners 3-PS; Figure S4: Meta-analysis of efficacy: WFIRS-P; Figure S5: Meta-analysis of safety: AEs; Figure S6: Meta-analysis of safety: SAEs; Figure S7: Subgroup analysis of efficacy: ADHD-RS-5; Figure S8: Subgroup analysis of efficacy: Number of patients with increased CGI-I score; Figure S9: Subgroup analysis of efficacy: Conners 3-PS; Figure S10: Subgroup analysis of efficacy: WFIRS-P; Figure S11: Subgroup analysis of safety: AEs; Figure S12: Subgroup analysis of safety: SAEs; Table S1: Inclusion, Exclusion criteria, Study design and Outcome assessments of the Included Studies; Table S2: Search strategy Table S1.
