# Supplementary material for: Efficacy and Safety of SPN-812 (Extended-Release Viloxazine) in Children and Adolescents with Attention-Deficit/Hyperactivity Disorder: A Systematic Review and Meta-Analysis
Source: Brain Sci. 2023 Nov 24;13(12):1627. doi: 10.3390/brainsci13121627 (PMC10742293; doi:10.3390/brainsci13121627)

Efficacy and Safety of SPN-812 (Extended-Release Viloxazine) in Children and Adolescents with Attention-Deficit/Hyperactivity Disorder: A Systematic Review and Meta-Analysis

Xin Tan [1,†], Yuejuan Xu [2,†], Shixin Wang [3], Jiaxuan Li [3], Chunxia Hu [2], Zhouqing Chen [3], Qingzhang Cheng [1,*] and Zhong Wang [3,4,*]

[1] Department of Neurology, The Affiliated Suzhou Hospital of Nanjing Medical University, Suzhou Municipal Hospital, Suzhou 215000, China; xintan862968596@163.com

[2] Department of Respiratory Medicine, Children's Hospital of Wujiang District, Children's Hospital of Soochow University, Suzhou 215025, China; xuyuejuan96@163.com (Y.X.); huchunxia780701@163.com (C.H.)

[3] Department of Neurosurgery& Brain and Nerve Research Laboratory, The First Affiliated Hospital of Soochow University, Suzhou 215006, China; wsx9036@163.com (S.W.); lijiaxuan1622@163.com (J.L.); zqchen6@163.com (Z.C.)

[4] Department of Neurosurgery, The First Affiliated Hospital of Soochow University, 188 Shizi Street, Suzhou 215006, China

* Correspondence: cqz202301@163.com (Q.C.); wangzhong761@163.com (Z.W.)

† These authors contributed equally to this work.

Figure legend

Figure S1: Meta-analysis of efficacy: ADHD-RS-5.

Figure S2: Meta-analysis of efficacy: Number of patients with increased CGI-I score.

Figure S3: Meta-analysis of efficacy: Conners 3-PS.

Figure S4: Meta-analysis of efficacy: WFIRS-P.

Figure S5: Meta-analysis of safety: AEs.

Figure S6: Meta-analysis of safety: SAEs.

Figure S7: Subgroup analysis of efficacy: ADHD-RS-5.

Figure S8: Subgroup analysis of efficacy: Number of patients with increased CGI-I score.

Figure S9: Subgroup analysis of efficacy: Conners 3-PS.

Figure S10: Subgroup analysis of efficacy: WFIRS-P.

Figure S11: Subgroup analysis of safety: AEs.

Figure S12: Subgroup analysis of safety: SAEs.

Table S1: Inclusion, Exclusion criteria, Study design and Outcome assessments of the Included Studies.

Table S2: Search strategy

Table S1: Inclusion, exclusion criteria, study design and outcome assessments of the included studies

**Trails**	**Nasser A et al 2021 (NCT03247556)**
Inclusion Criteria	Patients were eligible for inclusion in the trial if they were healthy male or female subjects, 12-17 years of age; diagnosis of ADHD according to the DSM-5, confirmed with MINI-KID; ADHD-RS-5 score at of them least 28; CGI-S score of them at least 4 at screening; weight of them at least 35 kg; free of medication for the treatment of ADHD for at least one week prior to randomization and agreement to remain so throughout the study; considered medically healthy by the Investigator via assessment of physical examination, medical history, clinical laboratory tests, vital signs, and electrocardiogram; written informed consent obtained from the subject's parent or legal representative and informed assent from the subject, if applicable; FOCP must be either sexually inactive (abstinent) or, if sexually active, must agree to use one of the following acceptable birth control methods beginning 30 days prior to the first dose, throughout the study.
Exclusion Criteria	Patients were excluded from the trial if they had current diagnosis of major psychiatric disorders; current diagnosis of major neurological disorders; current diagnosis of significant systemic disease; evidence of suicidality (defined as either active suicidal plan/intent or active suicidal thoughts, or more than one lifetime suicide attempt) within the six months before Screening or at Screening; BMI greater than 95th percentile for the appropriate age and gender; history of an allergic reaction to viloxazine or related drugs; any food allergy, intolerance, restriction or special diet that, in the opinion of the Investigator, could contraindicate the subject's participation in this study; received any investigational drug within the longer of 30 days or 5 half-lives prior to Day 1 dosing of SM; any reason, which, in the opinion of the Investigator, would prevent the subject from participating in the study; positive drug screen at the Screening Visit; pregnancy or refusal to practice abstinence or acceptable birth control during the study (for female subjects of childbearing potential).
Study Design	A multicenter, randomized, double-blind, placebo-controlled study to assess the efficacy and safety of SPN-812 (400mg and 600mg) as a monotherapy for the treatment of adolescents 12-17 years old with ADHD.
Efficacy Outcomes	The primary efficacy endpoint was CFB at EOS in the ADHD-RS-5 Total score. Key secondary endpoints included: CGI-I score at EOS and CFB at EOS in the Conners 3-PS Composite T-score and WFIRS-P Total average score.
Safety Outcomes	Adverse effects and severe adverse effects.

Trails	Johnson et al 2019<br>(NCT02633527)
Inclusion Criteria	Eligible patients were 6 to 12 years with a diagnosis of ADHD and to be deemed medically healthy through assessment of medical history, physical examination, clinical laboratory tests, and ECG. Participants, with a body weight of at least 20 kg, were required to have a minimum score of 26 on the ADHD-RS-IV and a minimum score of 4 on the CGI-S at Baseline. They also had to be free of ADHD medication for at least 1 week prior to Baseline. Participants could not take any ADHD medication or concomitant medication during the study period, with the exception of over the-counter agents such as nutritional supplements, common transient treatments (e.g., ibuprofen or acetaminophen), or other select concomitant medications including selective β2 -adrenoreceptor agonists (25 participants, 11.5% of the safety population).
Exclusion Criteria	Patients were excluded from the trial if they had a history or presence of neuropsychiatric disease other than ADHD as the primary diagnosis (i.e., the condition most impacting the child), including major depressive disorder, bipolar disorder, personality disorder, Tourette disorder, pervasive developmental disorder, obsessive compulsive disorder, post-traumatic stress disorder, or any other anxiety disorder or psychosis not otherwise specified. In addition, a history or presence of systemic disease including cardiovascular, pulmonary, hepatic, renal, hematologic, gastrointestinal, endocrine, immunologic, dermatologic, or other neurologic or psychiatric disease led to exclusion from the study. Additional exclusion criteria included evidence of suicidal attempt or ideation 6 months<br>prior to screening or at screening
Study Design	In this 8-week study, participants were randomized to placebo or SPN-812 100, 200, 300, or 400 mg/day, which is randomized, double-blind.
Efficacy Outcomes	The primary efficacy outcome measure was the change from Baseline to EOS in the ADHD-RS-IV total score. The secondary efficacy endpoints included change from Baseline in the CGI-S and CGI-I scores
Safety Outcomes	Adverse effects.

**Trails**	**Nasser A et al 2021 (NCT03247517)**
Inclusion Criteria	Study candidates (12–17 years of age, weight of ≥35 kg) were eligible to participate if they had a primary diagnosis of ADHD per the Diagnostic and Statistical Manual of Mental Disorders (Fifth Edition; American Psychiatric Association, 2013), confirmed via the Mini International Neuropsychiatric Interview for Children and Adolescents; an ADHD-RS-5 Total score ≥28 and a CGI-S score ≥4 (ie, overall illness severity of moderate or greater) at screening. Subjects were required to refrain from taking other ADHD medications for a minimum of 1 week before randomization and for the study duration. Subjects were eligible to participate if they were considered medically healthy by the study investigator via assessment of physical examination, medical history, clinical laboratory tests, vital signs, and ECG. Females of childbearing potential had to either be sexually inactive (abstinent) or agree to use one of the acceptable birth control methods beginning 30 days before the first dose and throughout the study.
Exclusion Criteria	Patients were excluded from the trial if they had a current diagnosis of a major psychiatric disorder, a major neurological disorder (including seizures or a history of seizure disorder within the immediate family, or a history of seizure-like events), a significant systemic disease, evidence of suicidality (defined as active suicidal plan or thoughts, or more than one lifetime suicide attempt) within 6 months of or at screening, and/or a body mass index >95th percentile for the appropriate age and sex, a history of intolerance or allergic reaction to viloxazine or its excipients, received any investigational drug within 30 days or 5 half-lives before first dosing of SM, or had any reason that in the opinion of the site investigator contraindicated participation. Other exclusion criteria were positive drug screen at the screening visit (a positive test result for amphetamines at screening was allowed for subjects receiving a prescription-stimulant ADHD medication and not responding to treatment—the subject was required to discontinue the stimulant for the study, beginning at least one week before the baseline visit), pregnancy, breastfeeding, or refusal to practice abstinence or acceptable birth control during the study (for females of childbearing potential).
Study Design	This trial was a multicenter, randomized, double-blind, placebo-controlled, 3-arm, parallel group trial in patients with ADHD.
Efficacy Outcomes	The primary efficacy end point was CFB at the EOS in ADHD Rating Scale-5 Total score. Key secondary end points were Clinical Global Impression—Improvement score at EOS, CFB at EOS in Conners 3—Parent Short Form Composite T-score, and CFB at EOS in Weiss Functional Impairment Rating Scale—Parent Total average score.
Safety Outcomes	Adverse events and serious adverse events.

**Trails**	**Nasser A et al 2020 (NCT03247530)**
Inclusion Criteria	Children (6-11 years of age at screening) were eligible to participate in the study if they had a primary diagnosis of ADHD as defined according to the DSM-5, which was confirmed by the MINI-KID, an ADHD-RS-5 Total score⩾28 at screening (visit 1) and baseline (visit 2), and a CGI-S score⩾4 (ie, moderate or greater overall illness severity) at screening (visit 1). Subjects were required to refrain from taking ADHD medications (other than the study medication) starting at least 1 week before randomization and throughout the study until EOS.
Exclusion Criteria	Subjects were not eligible to participate if they had a current diagnosis of a major psychiatric/ neurologic disorder other than ADHD (excluding oppositional defiant disorder, or major depressive disorder if the subject was free of major depressive episodes both currently and for the 6 months before screening), significant systemic disease, a history of allergic reaction to viloxazine or its excipients, any food allergy or intolerance that contraindicated trial participation, and/or evidence of suicidality within 6 months of screening.
Study Design	This study was a randomized, double blind, placebo-controlled 6-week trial to assess the efficacy and safety of once-daily 100- and 200-mg SPN-812 in the treatment of ADHD in male and female children 6-11 years of age.
Efficacy Outcomes	The primary efficacy endpoint of this trial was the CFB in the ADHD-RS-5 Total score at EOS (week 6). There were 3 key secondary endpoints: CGI-I score at EOS, CFB in the Conners 3—PS Composite T-score at EOS, and CFB in the WFIRS—P Total average score at EOS. Additional secondary endpoints included the CFB at EOS in each ADHD-RS-5 subscale (Inattention and Hyperactivity/Impulsivity); the 50% responder rate per the ADHD-RS-5 (defined as the proportion of subjects who exhibit a 50% reduction [improvement] in CFB ADHD-RS-5 Total score); responder rate per the CGI-I (or categorical CGI-I; proportion of subjects categorized as “improved,” which is defined as a subject who had a CGI-I score of 1 [“very much improved”] or 2 [“much improved”]) by visit; the CFB in the PSI-4-SF Total score at EOS; and the CFB in the Conners 3—SRS Composite T-score at EOS. Other endpoints included individual CFB at EOS in the content scale T-score for both the Conners 3—PS and Conners 3—SRS and CFB at EOS in the domain average score for WFIRS—P.
Safety Outcomes	Adverse effects and severe adverse effects.

Trails	Nasser A et al 2021 (NCT03247543)
Inclusion Criteria	Male and female children with ADHD who were aged 6-11 years and having a body weight of 20 kg were eligible for inclusion in the study. The key inclusion criteria were a primary diagnosis of ADHD as defined in the DSM-5, confirmed using the Mini International Neuropsychiatric Interview for Children and Adolescents, and an ADHD-RS-5 score of ⩾28 and a CGI-S score of ⩾4 at screening. Patients were eligible for participation if they were considered medically healthy by the study investigator via assessment of physical examination, medical history, clinical laboratory tests, vital sign measurement, and ECG. Eligible females of childbearing potential were either sexually inactive (abstinent) or agreed to use an acceptable method of birth control beginning 30 days prior to the administration of the first dose and throughout the study.
Exclusion Criteria	Exclusion criteria were a current diagnosis of a major psychiatric or neurologic disorder (including<br>seizures or a history of seizure disorder within the immediate family, or a history of seizure-like events); a significant systemic disease; evidence of suicidality (defined as active suicidal plan/intent, active suicidal thoughts, or more than 1 lifetime suicide attempt) within 6 months of screening; a body mass index in the >95th percentile for age and sex; history of an allergic reaction to viloxazine or related drugs; and/or food allergy, intolerance, or restricted or special diet that, in the opinion of the site investigator, might have precluded the patient's participation in the study. Patients were excluded from the study if they received any investigational drug within the longer of 30 days or 5 half-lives prior to day 1 of administration of SM or had a positive drug test result at screening.
Study Design	This study was randomized, double-blind, placebo-controlled trial to evaluated the efficacy and tolerability of SPN-812 200 and 400 mg once daily in children 6-11 years of age with ADHD.
Efficacy Outcomes	The primary efficacy endpoint was the CFB in ADHD-RS-5 Total score at EOS. Key secondary endpoints included CGI-I score at EOS, CFB in Conners 3-PS composite T score at EOS, and CFB in WFIRS-P Total average score at EOS.
Safety Outcomes	Adverse events and serious adverse events.

ADHD: Attention Deficit/Hyperactivity Disorder; Diagnostic and Statistical Manual of Mental Disorders-5 (DSM-5); Mini International Neuropsychiatric Interview for Children and Adolescents (MINI-KID); females of childbearing potential (FOCP); Body Mass Index (BMI); study medication (SM); change from baseline (CFB); end of study (EOS); Attention Deficit/Hyperactivity Disorder Rating Scale-5 (ADHD-RS-5); Clinical Global Impression-Improvement (CGI-I); Clinical Global Impression-Severity (CGI-S); Conners 3-Parent Short Form (Conners 3-PS); Weiss Functional Impairment Rating Scale-Parent (WFIRS-P); electrocardiogram (ECG); treatment-emergent adverse events (TEAEs); Columbia-Suicide Severity Rating Scale (C-SSRS).

Table S2:Search strategy

Search Date: August 30, 2023

Medline：

Search	Query	Results
#1	"Attention Deficit Disorder with Hyperactivity"[MeSH Terms]	35,140
#2	"attention deficit disorders with hyperactivity"[Title/Abstract] OR "ADHD"[Title/Abstract] OR "attention deficit hyperactivity disorder"[Title/Abstract] OR "hyperkinetic syndrome"[Title/Abstract] OR (("syndrom"[All Fields] OR "syndromal"[All Fields] OR "syndromally"[All Fields] OR "Syndrome"[MeSH Terms] OR "Syndrome"[All Fields] OR "syndromes"[All Fields] OR "syndrome s"[All Fields] OR "syndromic"[All Fields] OR "syndroms"[All Fields]) AND "Hyperkinetic"[Title/Abstract]) OR "attention deficit hyperactivity disorder"[Title/Abstract] OR "attention deficit hyperactivity disorders"[Title/Abstract] OR "deficit hyperactivity disorder attention"[Title/Abstract] OR "deficit hyperactivity disorders attention"[Title/Abstract] OR "disorder attention deficit hyperactivity"[Title/Abstract] OR "disorders attention deficit hyperactivity"[Title/Abstract] OR "ADDH"[Title/Abstract] OR "attention deficit hyperactivity disorders"[Title/Abstract] OR "attention deficit disorder"[Title/Abstract] OR "attention deficit disorders"[Title/Abstract] OR "deficit disorder attention"[Title/Abstract] OR "deficit disorders attention"[Title/Abstract] OR "disorder attention deficit"[Title/Abstract] OR "disorders attention deficit"[Title/Abstract] OR "brain dysfunction minimal"[Title/Abstract] OR (("dysfunctional"[All Fields] OR "dysfunctionals"[All Fields] OR "dysfunctioning"[All Fields] OR "dysfunctions"[All Fields] OR "physiopathology"[MeSH Subheading] OR "physiopathology"[All Fields] OR "Dysfunction"[All Fields]) AND "minimal brain"[Title/Abstract]) OR "minimal brain dysfunction"[Title/Abstract]	43,832
#3	#1 OR #2	50,443
#4	"Viloxazine"[MeSH Terms]	248
#5	"SPN-812"[Title/Abstract] OR "ici 58 834"[Title/Abstract] OR "ici58 834"[Title/Abstract] OR "viloxazine hydrochloride"[Title/Abstract] OR ((("Viloxazine"[MeSH Terms] OR "Viloxazine"[All Fields] OR ("Viloxazine"[All Fields] AND "Hydrochloride"[All Fields]) OR "viloxazine hydrochloride"[All Fields]) AND "R"[All Fields]) AND "isomer"[Title/Abstract]) OR "Vivalan"[Title/Abstract] OR ((("viloxazin"[All Fields] OR "Viloxazine"[MeSH Terms] OR "Viloxazine"[All Fields]) AND ("oxalates"[MeSH Terms] OR "oxalates"[All Fields] OR "oxalate"[All Fields] OR "oxalic"[All Fields])) AND "1 1"[Title/Abstract]) OR (("viloxazin"[All Fields] OR "Viloxazine"[MeSH Terms] OR "Viloxazine"[All Fields]) AND "isomer"[Title/Abstract]) OR ((("viloxazin"[All Fields] OR "Viloxazine"[MeSH Terms] OR "Viloxazine"[All Fields]) AND "R"[All Fields]) AND "isomer"[Title/Abstract]) OR ((("viloxazin"[All Fields] OR "Viloxazine"[MeSH Terms] OR "Viloxazine"[All Fields]) AND "S"[All Fields]) AND "isomer"[Title/Abstract]) OR "Emovit"[Title/Abstract] OR ((("Viloxazine"[MeSH Terms] OR "Viloxazine"[All Fields] OR	72

#1	("Viloxazine"[All Fields] AND "Hydrochloride"[All Fields]) OR "viloxazine hydrochloride"[All Fields]) AND "S"[All Fields]) AND "isomer"[Title/Abstract])	
#6	#4 OR #5	263
#7	#3 AND #6	39
#8	"Randomized controlled trial"[Filter]	604,444
#9	#7 AND #8	24

Embass：

Search	Query	Results
#1	'Attention Deficit Disorder with Hyperactivity'/exp	79,978
#2	'Attention Deficit Disorders with Hyperactivity':ab,ti,kw	16
#3	'ADHD':ab,ti,kw	47,742
#4	'Attention Deficit Hyperactivity Disorder':ab,ti,kw	40,589
#5	'Hyperkinetic Syndrome':ab,ti,kw	430
#6	'Syndromes, Hyperkinetic':ab,ti,kw	1
#7	'Attention Deficit-Hyperactivity Disorder':ab,ti,kw	40,598
#8	'Attention Deficit-Hyperactivity Disorders':ab,ti,kw	629
#9	'Deficit-Hyperactivity Disorder, Attention':ab,ti,kw	29
#10	'Deficit-Hyperactivity Disorders, Attention':ab,ti,kw	1
#11	'Disorder, Attention Deficit-Hyperactivity':ab,ti,kw	674
#12	'Disorders, Attention Deficit-Hyperactivity':ab,ti,kw	371
#13	'ADDH':ab,ti,kw	202
#14	'Attention Deficit Hyperactivity Disorders':ab,ti,kw	628
#15	'Attention Deficit Disorder':ab,ti,kw	3,227
#16	'Attention Deficit Disorders':ab,ti,kw	401
#17	'Deficit Disorder, Attention':ab,ti,kw	66
#18	'Deficit Disorders, Attention':ab,ti,kw	1
#19	'Disorder, Attention Deficit':ab,ti,kw	792
#20	'Disorders, Attention Deficit':ab,ti,kw	447
#21	'Brain Dysfunction, Minimal':ab,ti,kw	1
#22	'Dysfunction, Minimal Brain':ab,ti,kw	0
#23	'Minimal Brain Dysfunction':ab,ti,kw	713
#24	#1 OR #2 OR #3 OR #4 OR #5 OR #6 OR #7 OR #8 OR #9 OR #10 OR #11 OR #12 OR #13 OR #14 OR #15 OR #16 OR #17 OR #18 OR #19 OR #20 OR #21 OR #22 OR #23	87,170
#25	'viloxazine'/exp	1,594
#26	'SPN-812':ab,ti,kw	34
#27	'ICI-58,834':ab,ti,kw	11
#28	'ICI58,834':ab,ti,kw	0
#29	'Viloxazine Hydrochloride':ab,ti,kw	31
#30	'Viloxazine Hydrochloride, (R)-Isomer':ab,ti,kw	0
#31	'Vivalan':ab,ti,kw	67
#32	'Viloxazine Oxalate (1:1)':ab,ti,kw	0
#33	'Viloxazine, (+-)-Isomer':ab,ti,kw	0
#34	'Viloxazine, (R)-Isomer':ab,ti,kw	0

#35	'Viloxazine, (S)-Isomer':ab,ti,kw	0
#36	'Emovit':ab,ti,kw	18
#37	'Viloxazine Hydrochloride, (S)-Isomer':ab,ti,kw	0
#38	#25 OR #26 OR #27 OR #28 OR #29 OR #30 OR #31 OR #32 OR #33 OR #34 OR #35 OR #36 OR #37	1,595
#39	#24 AND #38	129
#40	'random':ab,ti OR 'control':ab,ti OR 'double-blind':ab,ti	4,666,358
#41	#39 AND #40	104

Cochrane:

Search	Query	Results
#1	MeSH descriptor: [Attention Deficit Disorder with Hyperactivity] explode all trees	3,507
#2	("Attention Deficit Disorders with Hyperactivity"):ti,ab,kw OR (ADHD):ti,ab,kw OR ("Attention Deficit Hyperactivity Disorder"):ti,ab,kw OR ("hyperkinetic syndrome"):ti,ab,kw OR ("Syndromes, Hyperkinetic"):ti,ab,kw	7,197
#3	("Attention Deficit-Hyperactivity Disorder"):ti,ab,kw OR ("Attention Deficit-Hyperactivity Disorders"):ti,ab,kw OR ("Deficit-Hyperactivity Disorder, Attention"):ti,ab,kw OR ("Deficit-Hyperactivity Disorders, Attention"):ti,ab,kw OR ("Disorder, Attention Deficit-Hyperactivity"):ti,ab,kw	5,367
#4	("Disorders, Attention Deficit-Hyperactivity"):ti,ab,kw OR ("ADDH"):ti,ab,kw OR ("Attention Deficit Hyperactivity Disorders"):ti,ab,kw OR ("Attention Deficit Disorder"):ti,ab,kw OR ("Attention Deficit Disorders"):ti,ab,kw	6,731
#5	("Deficit Disorder, Attention"):ti,ab,kw OR ("Deficit Disorders, Attention"):ti,ab,kw OR ("Disorder, Attention Deficit"):ti,ab,kw OR ("Disorders, Attention Deficit"):ti,ab,kw OR ("Brain Dysfunction, Minimal"):ti,ab,kw	174
#6	("Dysfunction, Minimal Brain"):ti,ab,kw OR ("Minimal Brain Dysfunction"):ti,ab,kw	54
#7	#1 OR #2 OR #3 OR #4 OR #5 OR #6	7,479
#8	MeSH descriptor: [Viloxazine] explode all trees	53
#9	("SPN-812"):ti,ab,kw OR ("ICI-58,834"):ti,ab,kw OR ("ICI58,834"):ti,ab,kw OR ("Viloxazine Hydrochloride"):ti,ab,kw OR ("Viloxazine Hydrochloride, (R)-Isomer"):ti,ab,kw	50
#10	("Vivalan"):ti,ab,kw OR ("Emovit"):ti,ab,kw OR ("Viloxazine Hydrochloride, (S)-Isomer"):ti,ab,kw OR ("Viloxazine, (R)-Isomer"):ti,ab,kw OR ("Viloxazine, (S)-Isomer"):ti,ab,kw	16
#11	#8 OR #9 OR #10	101
#12	#7 AND #11	54

Clinicaltrials.gov：

Search	Query	Results

#1	Status: All studies, condition or disease: Attention Deficit Disorder with Hyperactivity, and Intervention/treatment: Viloxazine, Study type: interventional(Clinical Trial)	7

Figure S1 Meta-analysis of efficacy: ADHD-RS-5

	SPN-812			Placebo				Mean Difference
Study or Subgroup	Mean	SD	Total	Mean	SD	Total	Weight	IV, Random, 95% CI
**1.1.1 SPN-812 100mg**								
NCT02633527	-16.7	13.6	45	-10.5	13.9	24	17.9%	-6.20 [-13.03, 0.63]
NCT03247530	-16.6	14.1	147	-10.9	14.2	155	82.1%	-5.70 [-8.89, -2.51]
**Subtotal (95% CI)**			**192**			**179**	**100.0%**	**-5.79 [-8.68, -2.90]**
Heterogeneity: Tau² = 0.00; Chi² = 0.02, df = 1 (P = 0.90); I² = 0%								
Test for overall effect: Z = 3.92 (P < 0.0001)								
**1.1.2 SPN-812 200mg**								
NCT02633527	-18.4	14.1	46	-10.5	13.9	24	8.4%	-7.90 [-14.79, -1.01]
NCT03247517	-16	14.1	94	-11.4	14	104	26.1%	-4.60 [-8.52, -0.68]
NCT03247530	-17.7	14.1	158	-10.9	14.2	155	40.8%	-6.80 [-9.94, -3.66]
NCT03247543	-17.6	14.8	107	-11.7	14.6	97	24.6%	-5.90 [-9.94, -1.86]
**Subtotal (95% CI)**			**405**			**380**	**100.0%**	**-6.10 [-8.10, -4.09]**
Heterogeneity: Tau² = 0.00; Chi² = 1.03, df = 3 (P = 0.80); I² = 0%								
Test for overall effect: Z = 5.96 (P < 0.00001)								
**1.1.3 SPN-812 300mg**								
NCT02633527	-18.6	13.9	47	-10.5	13.9	24	100.0%	-8.10 [-14.93, -1.27]
**Subtotal (95% CI)**			**47**			**24**	**100.0%**	**-8.10 [-14.93, -1.27]**
Heterogeneity: Not applicable								
Test for overall effect: Z = 2.32 (P = 0.02)								
**1.1.4 SPN-812 400mg**								
NCT02633527	-19	14.2	44	-10.5	13.9	24	9.5%	-8.50 [-15.47, -1.53]
NCT03247517	-16.5	14	103	-11.4	14	104	31.7%	-5.10 [-8.91, -1.29]
NCT03247543	-17.5	15	97	-11.7	14.6	97	26.6%	-5.80 [-9.97, -1.63]
NCT03247556	-18.3	13.5	99	-13.2	13.5	96	32.1%	-5.10 [-8.89, -1.31]
**Subtotal (95% CI)**			**343**			**321**	**100.0%**	**-5.61 [-7.76, -3.46]**
Heterogeneity: Tau² = 0.00; Chi² = 0.81, df = 3 (P = 0.85); I² = 0%								
Test for overall effect: Z = 5.12 (P < 0.00001)								
**1.1.5 SPN-812 600mg**								
NCT03247556	-16.7	13.7	97	-13.2	13.5	96	100.0%	-3.50 [-7.34, 0.34]
**Subtotal (95% CI)**			**97**			**96**	**100.0%**	**-3.50 [-7.34, 0.34]**
Heterogeneity: Not applicable								
Test for overall effect: Z = 1.79 (P = 0.07)								

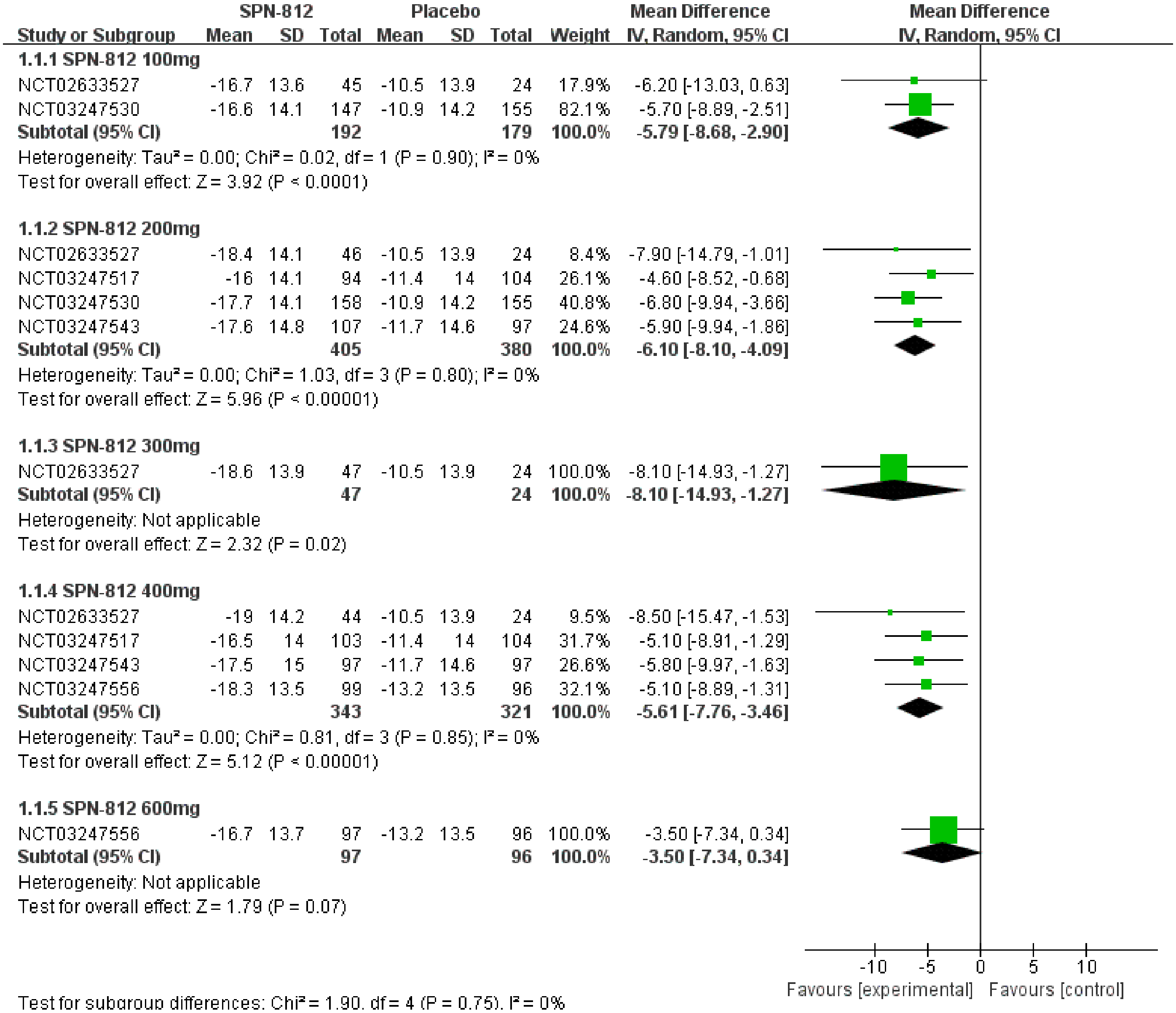

Test for subgroup differences: Chi² = 1.90, df = 4 (P = 0.75), I² = 0%

Figure S2 Meta-analysis of efficacy: number of patients with increased CGI-I score

Study or Subgroup	SPN-812 Events	SPN-812 Total	Placebo Events	Placebo Total	Weight	Risk Ratio IV, Random, 95% CI
**1.2.1 SPN-812 100mg**						
NCT03247530	66	147	46	155	100.0%	1.51 [1.12, 2.04]
**Subtotal (95% CI)**		**147**		**155**	**100.0%**	**1.51 [1.12, 2.04]**
Total events	66		46			
Heterogeneity: Not applicable						
Test for overall effect: Z = 2.69 (P = 0.007)						
**1.2.2 SPN-812 200mg**						
NCT03247517	48	94	33	104	28.6%	1.61 [1.14, 2.27]
NCT03247530	80	158	46	155	41.2%	1.71 [1.28, 2.27]
NCT03247543	51	107	34	97	30.1%	1.36 [0.97, 1.90]
**Subtotal (95% CI)**		**359**		**356**	**100.0%**	**1.57 [1.30, 1.88]**
Total events	179		113			
Heterogeneity: Tau² = 0.00; Chi² = 1.04, df = 2 (P = 0.59); I² = 0%						
Test for overall effect: Z = 4.78 (P < 0.00001)						
**1.2.3 SPN-812 400mg**						
NCT03247517	53	103	33	104	32.2%	1.62 [1.16, 2.28]
NCT03247543	46	97	34	97	31.5%	1.35 [0.96, 1.91]
NCT03247556	60	99	33	96	36.3%	1.76 [1.28, 2.43]
**Subtotal (95% CI)**		**299**		**297**	**100.0%**	**1.58 [1.30, 1.91]**
Total events	159		100			
Heterogeneity: Tau² = 0.00; Chi² = 1.27, df = 2 (P = 0.53); I² = 0%						
Test for overall effect: Z = 4.66 (P < 0.00001)						
**1.2.4 SPN-812 600mg**						
NCT03247556	46	97	33	96	100.0%	1.38 [0.98, 1.95]
**Subtotal (95% CI)**		**97**		**96**	**100.0%**	**1.38 [0.98, 1.95]**
Total events	46		33			
Heterogeneity: Not applicable						
Test for overall effect: Z = 1.82 (P = 0.07)						

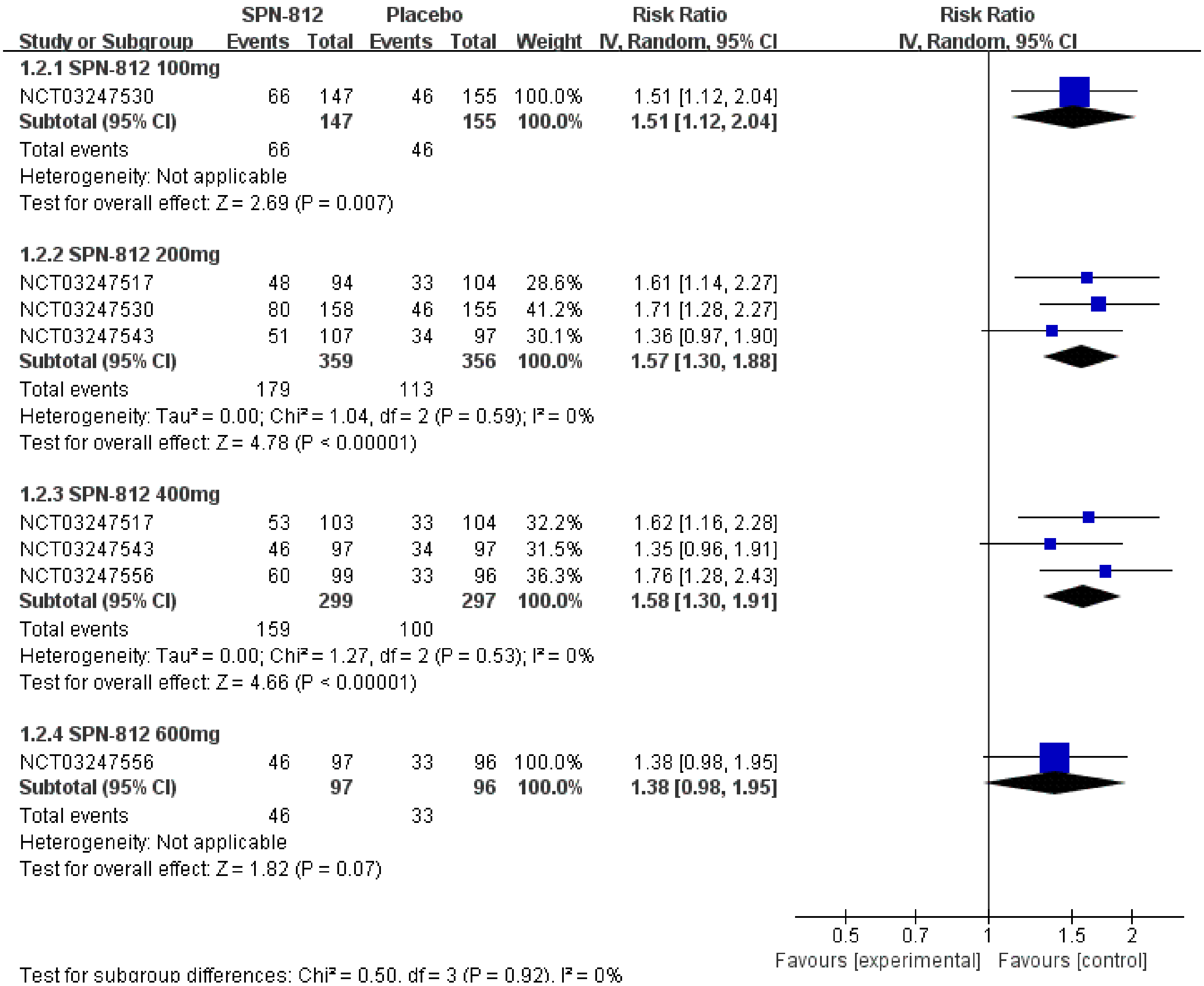

Test for subgroup differences: Chi² = 0.50, df = 3 (P = 0.92), I² = 0%

Figure S3 Meta-analysis of efficacy: Conners 3-PS

Study or Subgroup	SPN-812 Mean	SPN-812 SD	SPN-812 Total	Placebo Mean	Placebo SD	Placebo Total	Weight	Mean Difference IV, Random, 95% CI
**1.3.1 SPN-812 100mg**								
NCT03247530	-9.1	10.1	147	-4.8	10.1	155	100.0%	-4.30 [-6.58, -2.02]
**Subtotal (95% CI)**			**147**			**155**	**100.0%**	**-4.30 [-6.58, -2.02]**
Heterogeneity: Not applicable								
Test for overall effect: Z = 3.70 (P = 0.0002)								
**1.3.2 SPN-812 200mg**								
NCT03247517	-6.4	10.9	94	-5.7	10.6	104	29.0%	-0.70 [-3.70, 2.30]
NCT03247530	-9.2	10.3	158	-4.8	10.1	155	38.6%	-4.40 [-6.66, -2.14]
NCT03247543	-9.1	9.9	107	-5.3	9.8	97	32.5%	-3.80 [-6.51, -1.09]
**Subtotal (95% CI)**			**359**			**356**	**100.0%**	**-3.13 [-5.27, -1.00]**
Heterogeneity: Tau² = 1.75; Chi² = 3.92, df = 2 (P = 0.14); I² = 49%								
Test for overall effect: Z = 2.88 (P = 0.004)								
**1.3.3 SPN-812 400mg**								
NCT03247517	-8.6	10.9	103	-5.7	10.6	104	28.6%	-2.90 [-5.83, 0.03]
NCT03247543	-7.8	10.4	97	-5.3	9.8	97	30.3%	-2.50 [-5.34, 0.34]
NCT03247556	-7.5	8.7	99	-5.6	8.7	96	41.1%	-1.90 [-4.34, 0.54]
**Subtotal (95% CI)**			**299**			**297**	**100.0%**	**-2.37 [-3.93, -0.80]**
Heterogeneity: Tau² = 0.00; Chi² = 0.28, df = 2 (P = 0.87); I² = 0%								
Test for overall effect: Z = 2.96 (P = 0.003)								
**1.3.4 SPN-812 600mg**								
NCT03247556	-6.9	8.7	97	-5.6	8.7	96	100.0%	-1.30 [-3.75, 1.15]
**Subtotal (95% CI)**			**97**			**96**	**100.0%**	**-1.30 [-3.75, 1.15]**
Heterogeneity: Not applicable								
Test for overall effect: Z = 1.04 (P = 0.30)								

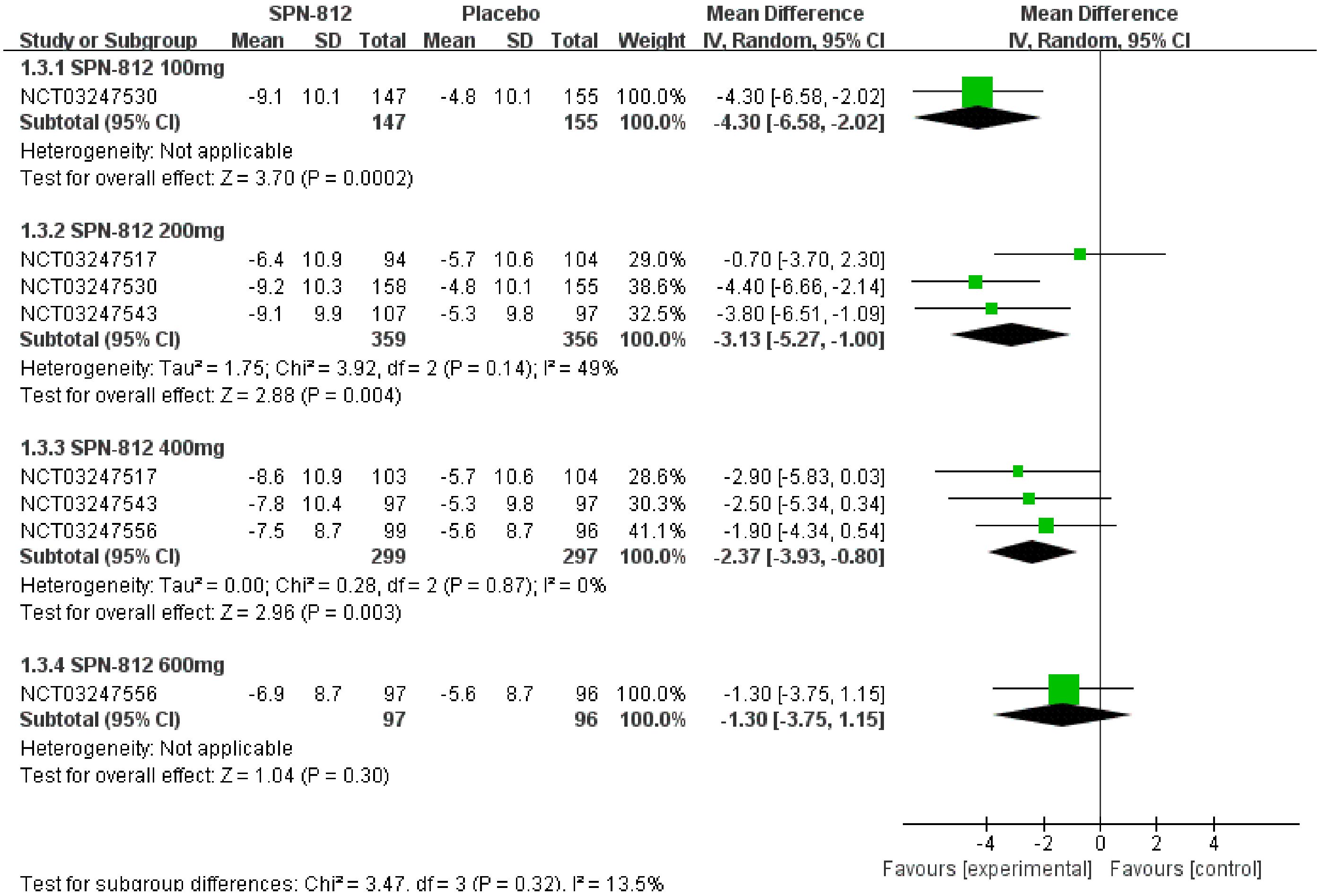

Test for subgroup differences: Chi² = 3.47, df = 3 (P = 0.32), I² = 13.5%

Figure S4 Meta-analysis of efficacy: WFIRS-P

	SPN-812			Placebo				Mean Difference
Study or Subgroup	Mean	SD	Total	Mean	SD	Total	Weight	IV, Random, 95% CI
**1.4.1 SPN-812 100mg**								
NCT03247530	-0.36	0.4	147	-0.22	0.4	155	100.0%	-0.14 [-0.23, -0.05]
**Subtotal (95% CI)**			**147**			**155**	**100.0%**	**-0.14 [-0.23, -0.05]**
Heterogeneity: Not applicable								
Test for overall effect: Z = 3.04 (P = 0.002)								
**1.4.2 SPN-812 200mg**								
NCT03247517	-0.27	0.4	94	-0.19	0.4	104	27.7%	-0.08 [-0.19, 0.03]
NCT03247530	-0.39	0.4	158	-0.22	0.4	155	43.8%	-0.17 [-0.26, -0.08]
NCT03247543	-0.35	0.4	107	-0.24	0.4	97	28.5%	-0.11 [-0.22, -0.00]
**Subtotal (95% CI)**			**359**			**356**	**100.0%**	**-0.13 [-0.19, -0.07]**
Heterogeneity: Tau² = 0.00; Chi² = 1.68, df = 2 (P = 0.43); I² = 0%								
Test for overall effect: Z = 4.28 (P < 0.0001)								
**1.4.3 SPN-812 400mg**								
NCT03247517	-0.31	0.4	103	-0.19	0.4	104	27.7%	-0.12 [-0.23, -0.01]
NCT03247543	-0.33	0.4	97	-0.24	0.4	97	26.0%	-0.09 [-0.20, 0.02]
NCT03247556	-0.32	0.3	99	-0.23	0.3	96	46.4%	-0.09 [-0.17, -0.01]
**Subtotal (95% CI)**			**299**			**297**	**100.0%**	**-0.10 [-0.16, -0.04]**
Heterogeneity: Tau² = 0.00; Chi² = 0.21, df = 2 (P = 0.90); I² = 0%								
Test for overall effect: Z = 3.36 (P = 0.0008)								
**1.4.4 SPN-812 600mg**								
NCT03247556	-0.23	0.3	97	-0.23	0.3	96	100.0%	0.00 [-0.08, 0.08]
**Subtotal (95% CI)**			**97**			**96**	**100.0%**	**0.00 [-0.08, 0.08]**
Heterogeneity: Not applicable								
Test for overall effect: Z = 0.00 (P = 1.00)								

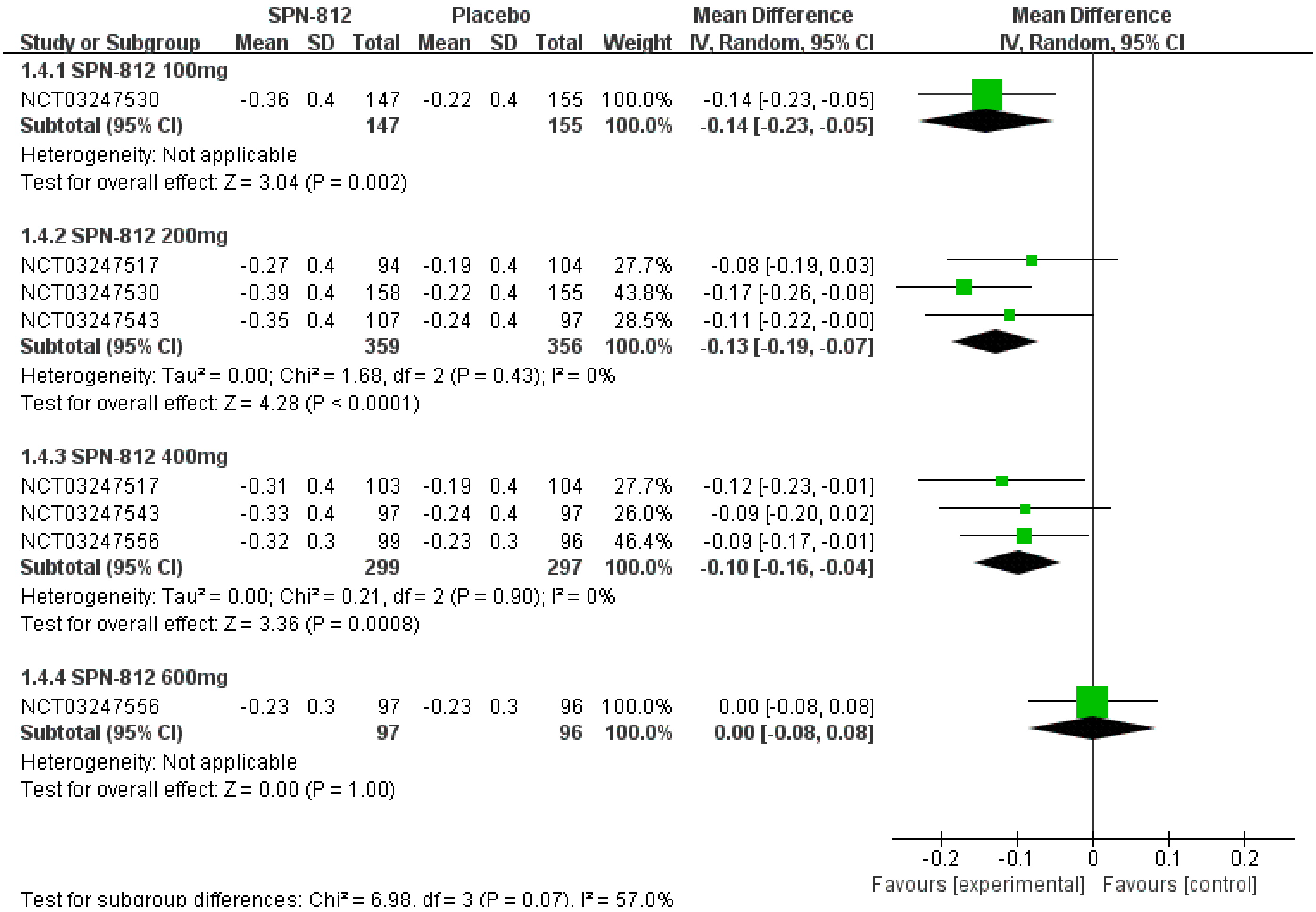

Test for subgroup differences: Chi² = 6.98, df = 3 (P = 0.07), I² = 57.0%

Figure S5 Meta-analysis of safety: AEs

	SPN-812		Placebo			Risk Ratio
Study or Subgroup	Events	Total	Events	Total	Weight	IV, Random, 95% CI
**1.5.1 SPN-812 100mg**						
NCT02633527	34	48	11	24	27.6%	1.55 [0.96, 2.48]
NCT03247530	74	154	47	159	72.4%	1.63 [1.22, 2.17]
**Subtotal (95% CI)**		**202**		**183**	**100.0%**	**1.60 [1.25, 2.05]**
Total events	108		58			
Heterogeneity: Tau² = 0.00; Chi² = 0.03, df = 1 (P = 0.86); I² = 0%						
Test for overall effect: Z = 3.74 (P = 0.0002)						
**1.5.2 SPN-812 200mg**						
NCT02633527	30	48	11	24	11.8%	1.36 [0.84, 2.22]
NCT03247517	43	99	38	104	23.4%	1.19 [0.85, 1.67]
NCT03247530	77	161	47	159	31.2%	1.62 [1.21, 2.16]
NCT03247543	56	107	47	103	33.5%	1.15 [0.87, 1.51]
**Subtotal (95% CI)**		**415**		**390**	**100.0%**	**1.31 [1.11, 1.56]**
Total events	206		143			
Heterogeneity: Tau² = 0.00; Chi² = 3.27, df = 3 (P = 0.35); I² = 8%						
Test for overall effect: Z = 3.14 (P = 0.002)						
**1.5.3 SPN-812 300mg**						
NCT02633527	33	48	11	24	100.0%	1.50 [0.93, 2.41]
**Subtotal (95% CI)**		**48**		**24**	**100.0%**	**1.50 [0.93, 2.41]**
Total events	33		11			
Heterogeneity: Not applicable						
Test for overall effect: Z = 1.67 (P = 0.09)						
**1.5.4 SPN-812 400mg**						
NCT02633527	35	49	11	24	18.3%	1.56 [0.97, 2.49]
NCT03247517	56	105	38	104	25.4%	1.46 [1.07, 1.99]
NCT03247543	58	100	47	103	27.5%	1.27 [0.97, 1.66]
NCT03247556	92	100	40	97	28.8%	2.23 [1.75, 2.85]
**Subtotal (95% CI)**		**354**		**328**	**100.0%**	**1.61 [1.21, 2.13]**
Total events	241		136			
Heterogeneity: Tau² = 0.06; Chi² = 10.12, df = 3 (P = 0.02); I² = 70%						
Test for overall effect: Z = 3.27 (P = 0.001)						
**1.5.5 SPN-812 600mg**						
NCT03247556	74	99	40	97	100.0%	1.81 [1.39, 2.36]
**Subtotal (95% CI)**		**99**		**97**	**100.0%**	**1.81 [1.39, 2.36]**
Total events	74		40			
Heterogeneity: Not applicable						
Test for overall effect: Z = 4.42 (P < 0.00001)						

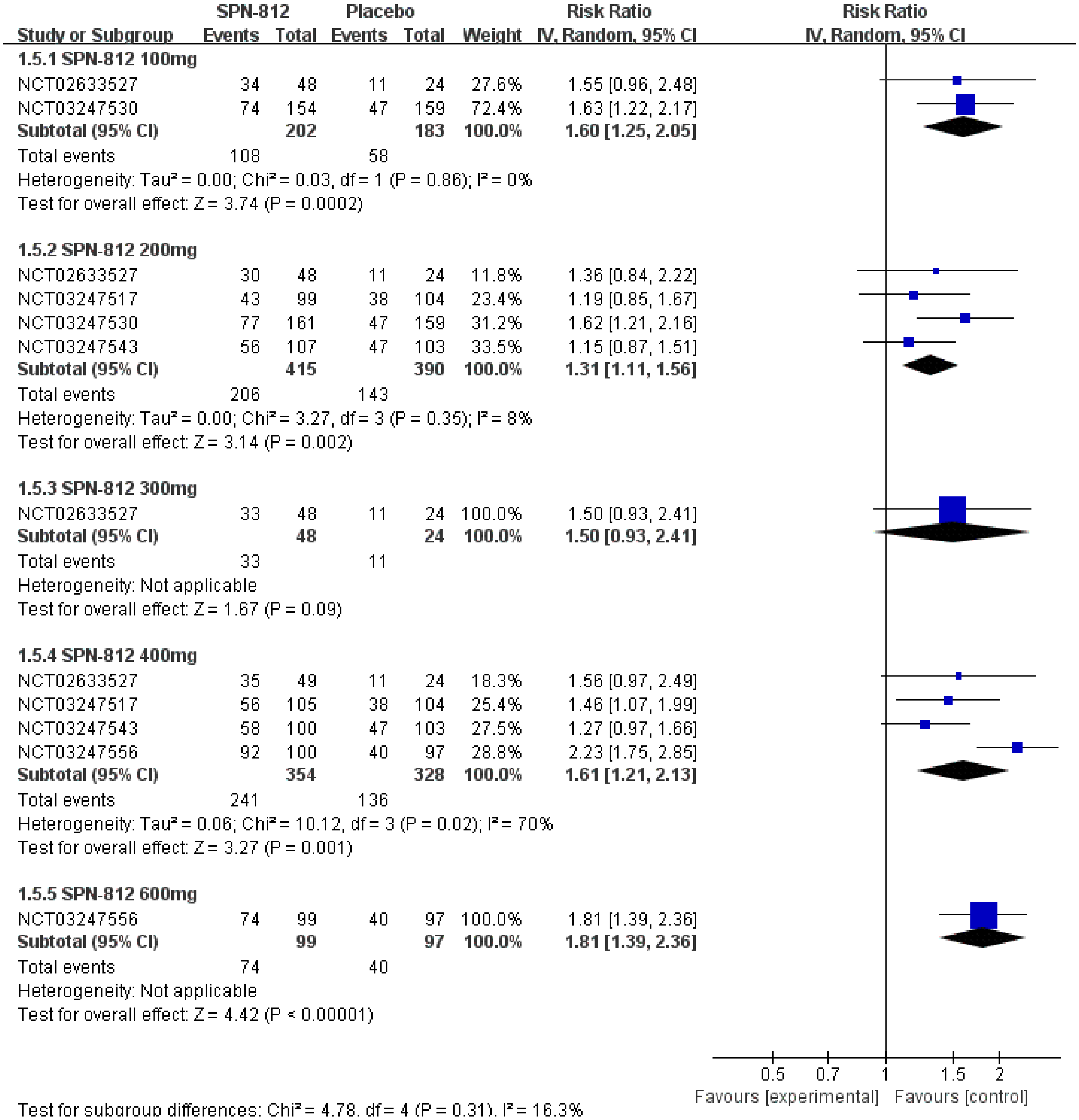

Test for subgroup differences: Chi² = 4.78, df = 4 (P = 0.31), I² = 16.3%

Figure S6 Meta-analysis of safety: SAEs

Study or Subgroup	SPN-812 Events	SPN-812 Total	Placebo Events	Placebo Total	Weight	Risk Ratio IV, Random, 95% CI
**1.6.1 SPN-812 100mg**						
NCT03247530	2	154	0	159	100.0%	5.16 [0.25, 106.64]
**Subtotal (95% CI)**		**154**		**159**	**100.0%**	**5.16 [0.25, 106.64]**
Total events	2		0			
Heterogeneity: Not applicable						
Test for overall effect: Z = 1.06 (P = 0.29)						
**1.6.2 SPN-812 200mg**						
NCT03247517	2	99	0	104	35.8%	5.25 [0.26, 108.01]
NCT03247530	1	161	0	159	32.1%	2.96 [0.12, 72.19]
NCT03247543	1	107	0	103	32.2%	2.89 [0.12, 70.11]
**Subtotal (95% CI)**		**367**		**366**	**100.0%**	**3.61 [0.59, 22.00]**
Total events	4		0			
Heterogeneity: Tau² = 0.00; Chi² = 0.09, df = 2 (P = 0.95); I² = 0%						
Test for overall effect: Z = 1.39 (P = 0.16)						
**1.6.3 SPN-812 400mg**						
NCT03247543	2	100	0	103	50.0%	5.15 [0.25, 105.92]
NCT03247556	2	100	0	97	50.0%	4.85 [0.24, 99.77]
**Subtotal (95% CI)**		**200**		**200**	**100.0%**	**5.00 [0.59, 42.40]**
Total events	4		0			
Heterogeneity: Tau² = 0.00; Chi² = 0.00, df = 1 (P = 0.98); I² = 0%						
Test for overall effect: Z = 1.47 (P = 0.14)						

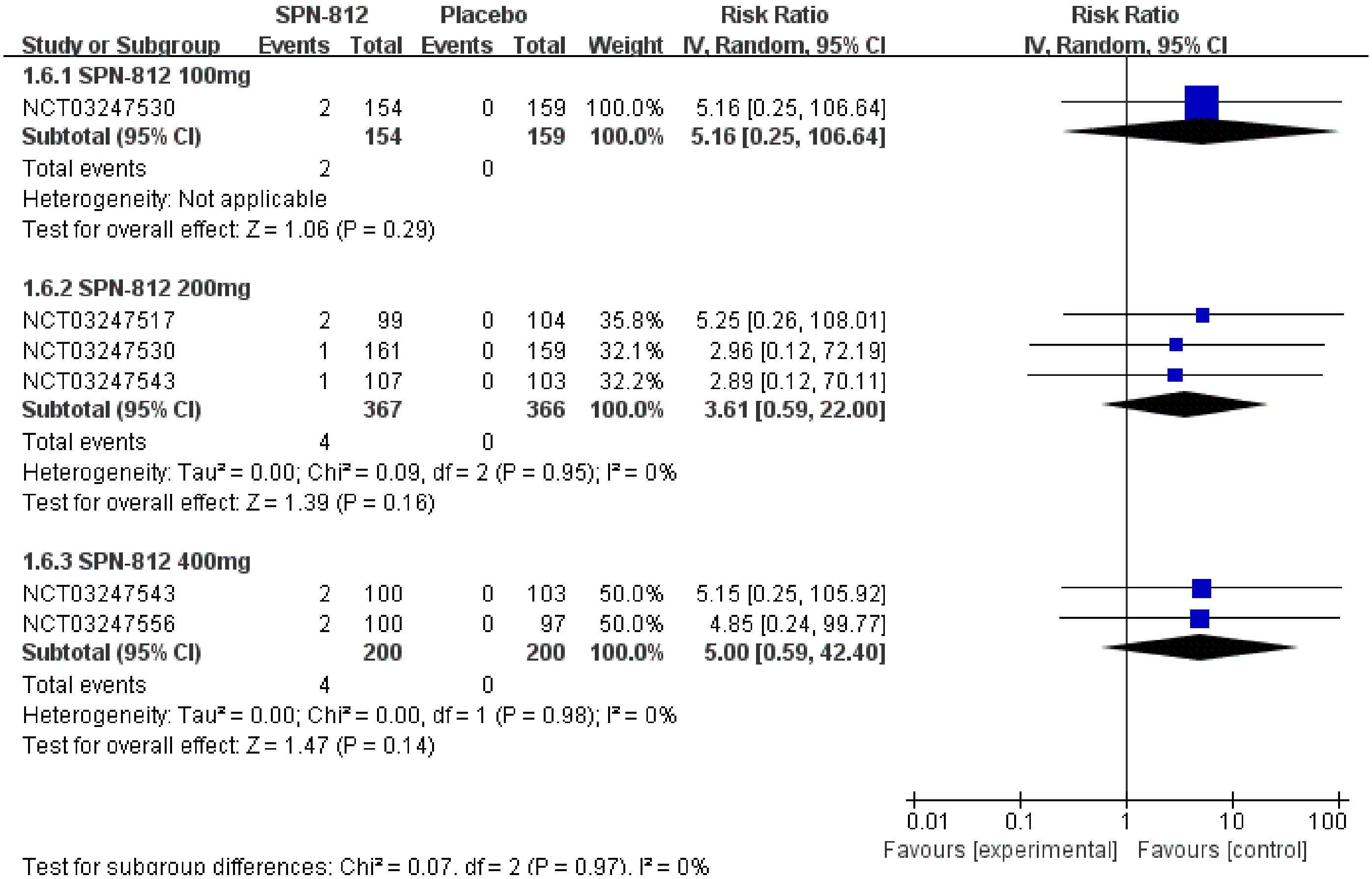

Test for subgroup differences: Chi² = 0.07, df = 2 (P = 0.97), I² = 0%

Figure S7 Subgroup analysis of efficacy: ADHD-RS-5

Study or Subgroup	SPN-812 Mean	SPN-812 SD	SPN-812 Total	Placebo Mean	Placebo SD	Placebo Total	Weight	Mean Difference IV, Random, 95% CI
**2.1.1 Children**								
NCT02633527	-18.2	13.9	182	-10.5	13.9	24	6.8%	-7.70 [-13.62, -1.78]
NCT03247530	-17.2	14.1	305	-10.9	14.2	155	31.5%	-6.30 [-9.04, -3.56]
NCT03247543	-17.6	14.9	204	-11.7	14.6	97	18.7%	-5.90 [-9.45, -2.35]
**Subtotal (95% CI)**			**691**			**276**	**57.0%**	**-6.33 [-8.37, -4.30]**
Heterogeneity: Tau² = 0.00; Chi² = 0.26, df = 2 (P = 0.88); I² = 0%								
Test for overall effect: Z = 6.10 (P < 0.00001)								
**2.1.2 Adolescents**								
NCT03247517	-16.3	14	197	-11.4	14	104	21.4%	-4.90 [-8.23, -1.57]
NCT03247556	-17.5	13.6	196	-13.2	13.5	96	21.6%	-4.30 [-7.60, -1.00]
**Subtotal (95% CI)**			**393**			**200**	**43.0%**	**-4.60 [-6.94, -2.25]**
Heterogeneity: Tau² = 0.00; Chi² = 0.06, df = 1 (P = 0.80); I² = 0%								
Test for overall effect: Z = 3.84 (P = 0.0001)								
**Total (95% CI)**			**1084**			**476**	**100.0%**	**-5.59 [-7.12, -4.05]**
Heterogeneity: Tau² = 0.00; Chi² = 1.53, df = 4 (P = 0.82); I² = 0%								
Test for overall effect: Z = 7.12 (P < 0.00001)								
Test for subgroup differences: Chi² = 1.20, df = 1 (P = 0.27), I² = 16.8%								

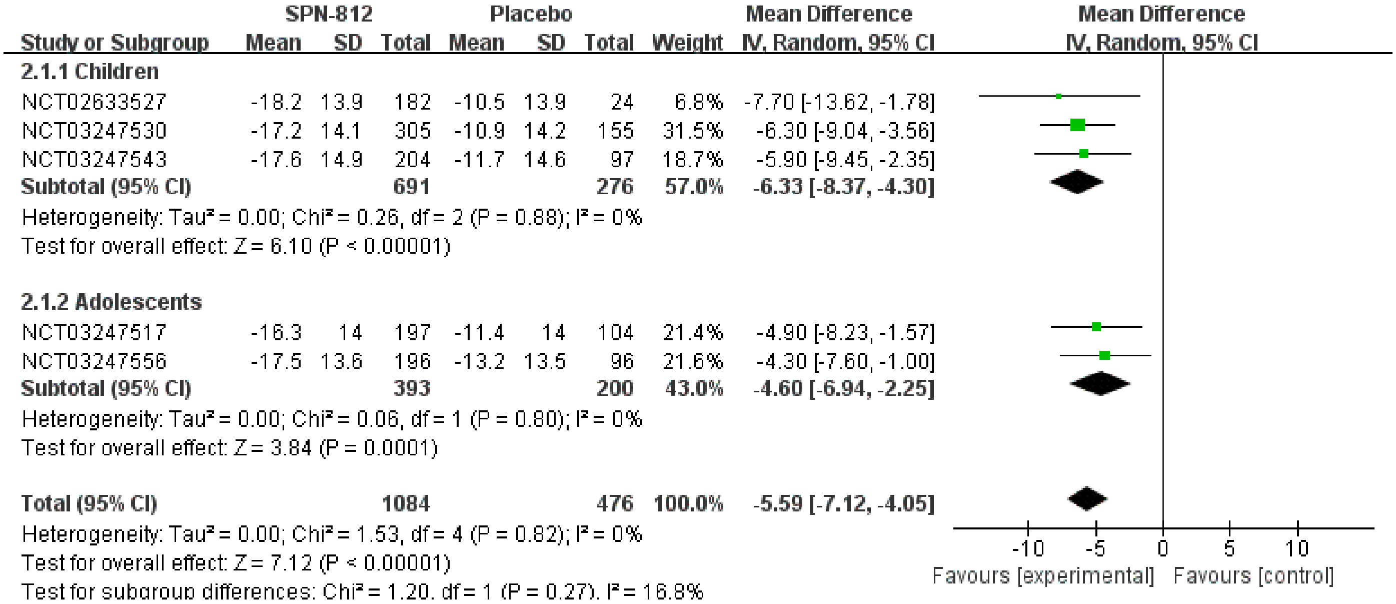

Figure S8 Subgroup analysis of efficacy: Number of patients with increased CGI-I score

Study or Subgroup	SPN-812 Events	SPN-812 Total	Placebo Events	Placebo Total	Weight	Risk Ratio IV, Random, 95% CI
**2.2.1 Children**						
NCT03247530	146	305	46	155	30.4%	1.61 [1.23, 2.11]
NCT03247543	97	204	34	97	23.4%	1.36 [1.00, 1.84]
**Subtotal (95% CI)**		**509**		**252**	**53.8%**	**1.50 [1.22, 1.83]**
Total events	243		80			
Heterogeneity: Tau² = 0.00; Chi² = 0.69, df = 1 (P = 0.41); I² = 0%						
Test for overall effect: Z = 3.90 (P < 0.0001)						
**2.2.2 Adolescents**						
NCT03247517	101	197	33	104	22.5%	1.62 [1.18, 2.21]
NCT03247556	106	196	33	96	23.7%	1.57 [1.16, 2.13]
**Subtotal (95% CI)**		**393**		**200**	**46.2%**	**1.59 [1.28, 1.98]**
Total events	207		66			
Heterogeneity: Tau² = 0.00; Chi² = 0.01, df = 1 (P = 0.90); I² = 0%						
Test for overall effect: Z = 4.18 (P < 0.0001)						
**Total (95% CI)**		**902**		**452**	**100.0%**	**1.54 [1.33, 1.79]**
Total events	450		146			
Heterogeneity: Tau² = 0.00; Chi² = 0.88, df = 3 (P = 0.83); I² = 0%						
Test for overall effect: Z = 5.70 (P < 0.00001)						
Test for subgroup differences: Chi² = 0.17, df = 1 (P = 0.68), I² = 0%						

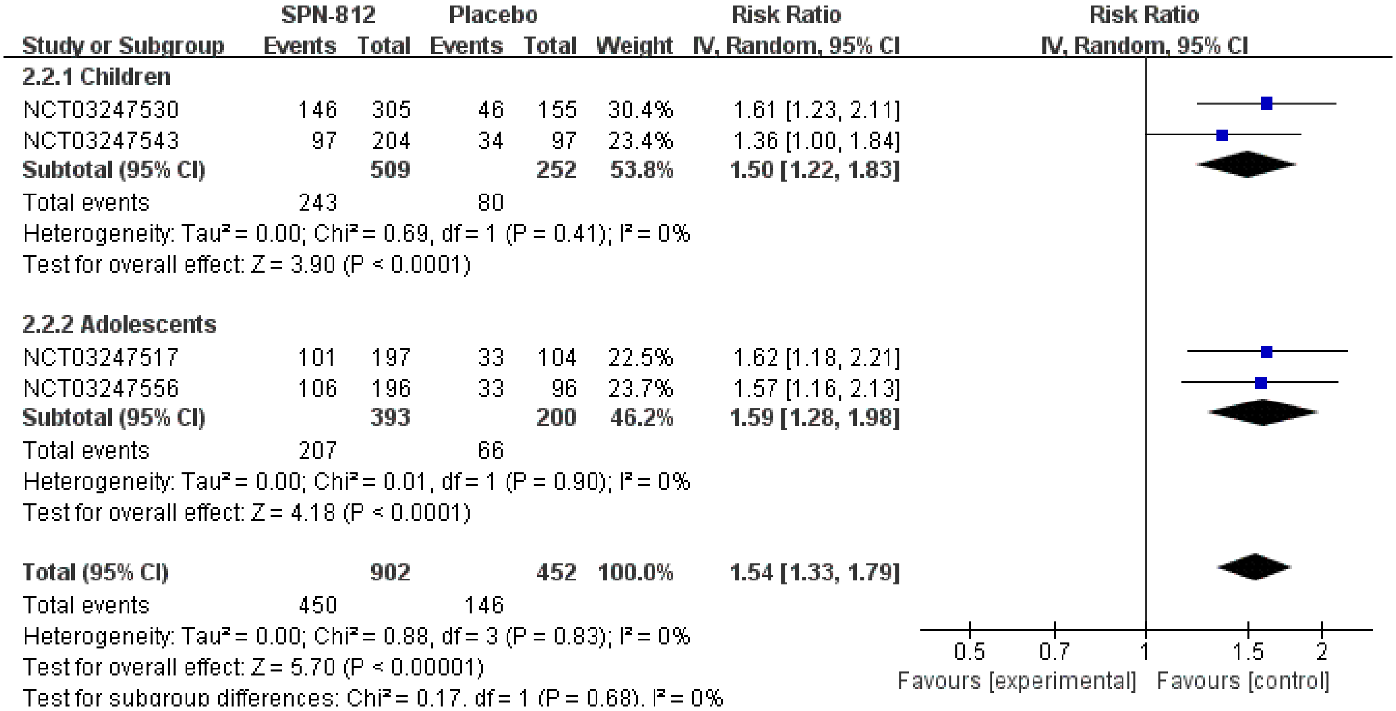

Figure S9 Subgroup analysis of efficacy: Conners 3-PS

Study or Subgroup	SPN-812 Mean	SPN-812 SD	SPN-812 Total	Placebo Mean	Placebo SD	Placebo Total	Weight	Mean Difference IV, Random, 95% CI
**2.3.1 Children**								
NCT03247530	-9.2	10.2	305	-4.8	10.1	155	29.7%	-4.40 [-6.36, -2.44]
NCT03247543	-8.5	10.1	204	-5.3	9.8	97	22.7%	-3.20 [-5.59, -0.81]
**Subtotal (95% CI)**			**509**			**252**	**52.5%**	**-3.92 [-5.43, -2.40]**
Heterogeneity: Tau² = 0.00; Chi² = 0.58, df = 1 (P = 0.45); I² = 0%								
Test for overall effect: Z = 5.07 (P < 0.00001)								
**2.3.2 Adolescents**								
NCT03247517	-7.6	10.9	197	-5.7	10.6	104	20.8%	-1.90 [-4.44, 0.64]
NCT03247556	-7.2	8.7	196	-5.6	8.7	96	26.8%	-1.60 [-3.72, 0.52]
**Subtotal (95% CI)**			**393**			**200**	**47.5%**	**-1.72 [-3.35, -0.09]**
Heterogeneity: Tau² = 0.00; Chi² = 0.03, df = 1 (P = 0.86); I² = 0%								
Test for overall effect: Z = 2.07 (P = 0.04)								
**Total (95% CI)**			**902**			**452**	**100.0%**	**-2.86 [-4.20, -1.51]**
Heterogeneity: Tau² = 0.58; Chi² = 4.34, df = 3 (P = 0.23); I² = 31%								
Test for overall effect: Z = 4.17 (P < 0.0001)								
Test for subgroup differences: Chi² = 3.73, df = 1 (P = 0.05), I² = 73.2%								

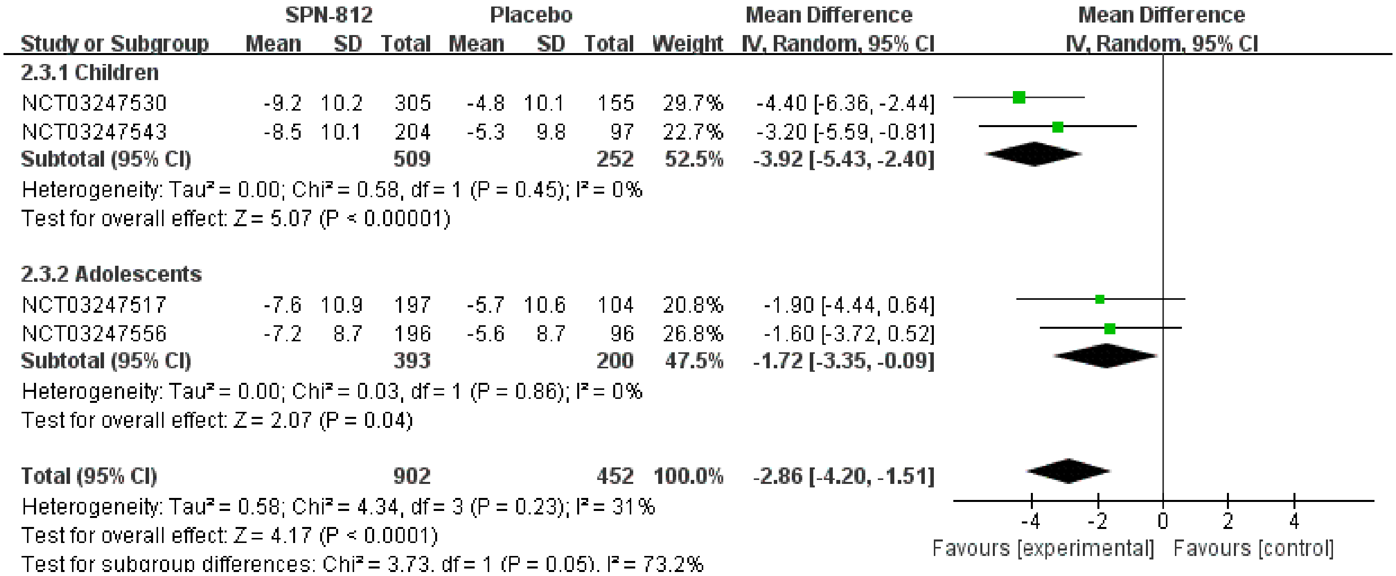

Figure S10 Subgroup analysis of efficacy: WFIRS-P

Study or Subgroup	SPN-812 Mean	SPN-812 SD	SPN-812 Total	Placebo Mean	Placebo SD	Placebo Total	Weight	Mean Difference IV, Random, 95% CI
**2.4.1 Children**								
NCT03247530	-0.38	0.4	305	-0.22	0.4	155	28.3%	-0.16 [-0.24, -0.08]
NCT03247543	-0.34	0.4	204	-0.24	0.4	97	20.3%	-0.10 [-0.20, -0.00]
**Subtotal (95% CI)**			**509**			**252**	**48.6%**	**-0.14 [-0.20, -0.08]**
Heterogeneity: Tau² = 0.00; Chi² = 0.90, df = 1 (P = 0.34); I² = 0%								
Test for overall effect: Z = 4.43 (P < 0.00001)								
**2.4.2 Adolescents**								
NCT03247517	-0.29	0.4	197	-0.19	0.4	104	20.9%	-0.10 [-0.20, -0.00]
NCT03247556	-0.28	0.3	196	-0.23	0.3	96	30.5%	-0.05 [-0.12, 0.02]
**Subtotal (95% CI)**			**393**			**200**	**51.4%**	**-0.07 [-0.13, -0.01]**
Heterogeneity: Tau² = 0.00; Chi² = 0.67, df = 1 (P = 0.41); I² = 0%								
Test for overall effect: Z = 2.32 (P = 0.02)								
**Total (95% CI)**			**902**			**452**	**100.0%**	**-0.10 [-0.15, -0.05]**
Heterogeneity: Tau² = 0.00; Chi² = 4.10, df = 3 (P = 0.25); I² = 27%								
Test for overall effect: Z = 4.04 (P < 0.0001)								
Test for subgroup differences: Chi² = 2.53, df = 1 (P = 0.11), I² = 60.5%								

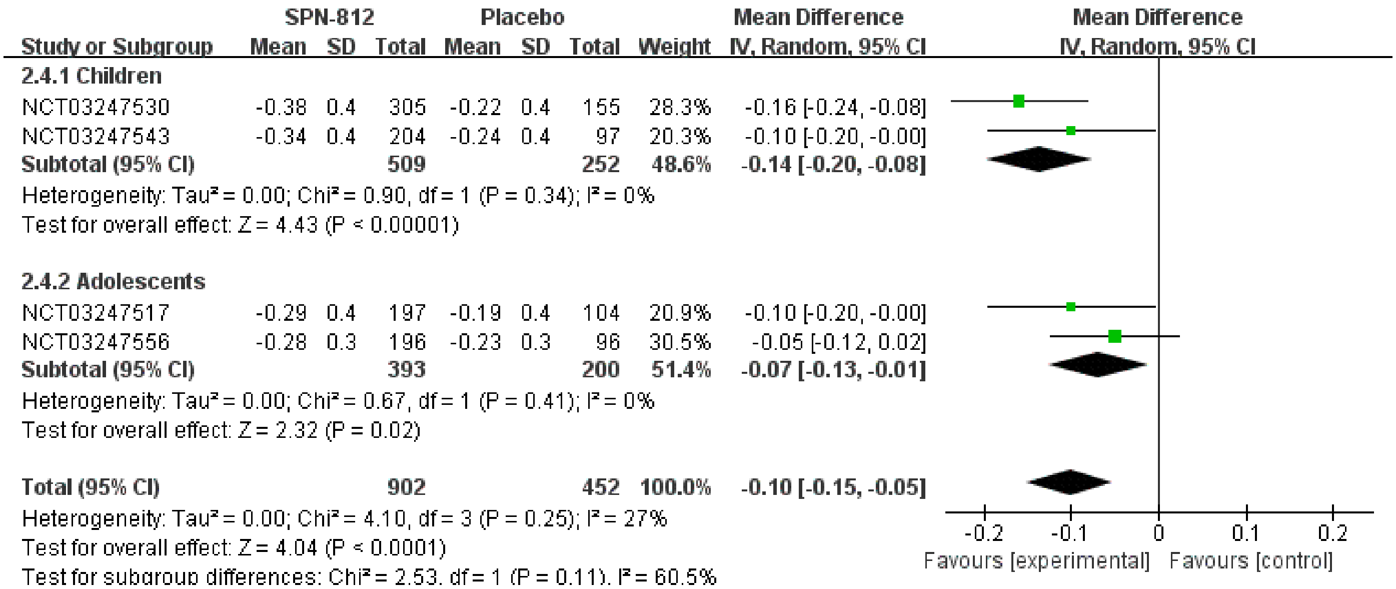

Figure S11 Subgroup analysis of safety: AEs

Study or Subgroup	SPN-812 Events	SPN-812 Total	Placebo Events	Placebo Total	Weight	Risk Ratio IV, Random, 95% CI
**2.5.1 Children**						
NCT02633527	132	193	11	24	12.7%	1.49 [0.96, 2.33]
NCT03247530	151	315	47	159	21.5%	1.62 [1.24, 2.12]
NCT03247543	114	207	47	103	22.9%	1.21 [0.95, 1.54]
**Subtotal (95% CI)**		**715**		**286**	**57.1%**	**1.40 [1.15, 1.71]**
Total events	397		105			
Heterogeneity: Tau² = 0.01; Chi² = 2.67, df = 2 (P = 0.26); I² = 25%						
Test for overall effect: Z = 3.35 (P = 0.0008)						
**2.5.2 Adolescents**						
NCT03247517	99	204	38	104	20.1%	1.33 [0.99, 1.78]
NCT03247556	166	199	40	97	22.8%	2.02 [1.58, 2.59]
**Subtotal (95% CI)**		**403**		**201**	**42.9%**	**1.65 [1.09, 2.49]**
Total events	265		78			
Heterogeneity: Tau² = 0.07; Chi² = 4.71, df = 1 (P = 0.03); I² = 79%						
Test for overall effect: Z = 2.39 (P = 0.02)						
**Total (95% CI)**		**1118**		**487**	**100.0%**	**1.52 [1.24, 1.85]**
Total events	662		183			
Heterogeneity: Tau² = 0.03; Chi² = 9.70, df = 4 (P = 0.05); I² = 59%						
Test for overall effect: Z = 4.09 (P < 0.0001)						
Test for subgroup differences: Chi² = 0.48, df = 1 (P = 0.49), I² = 0%						

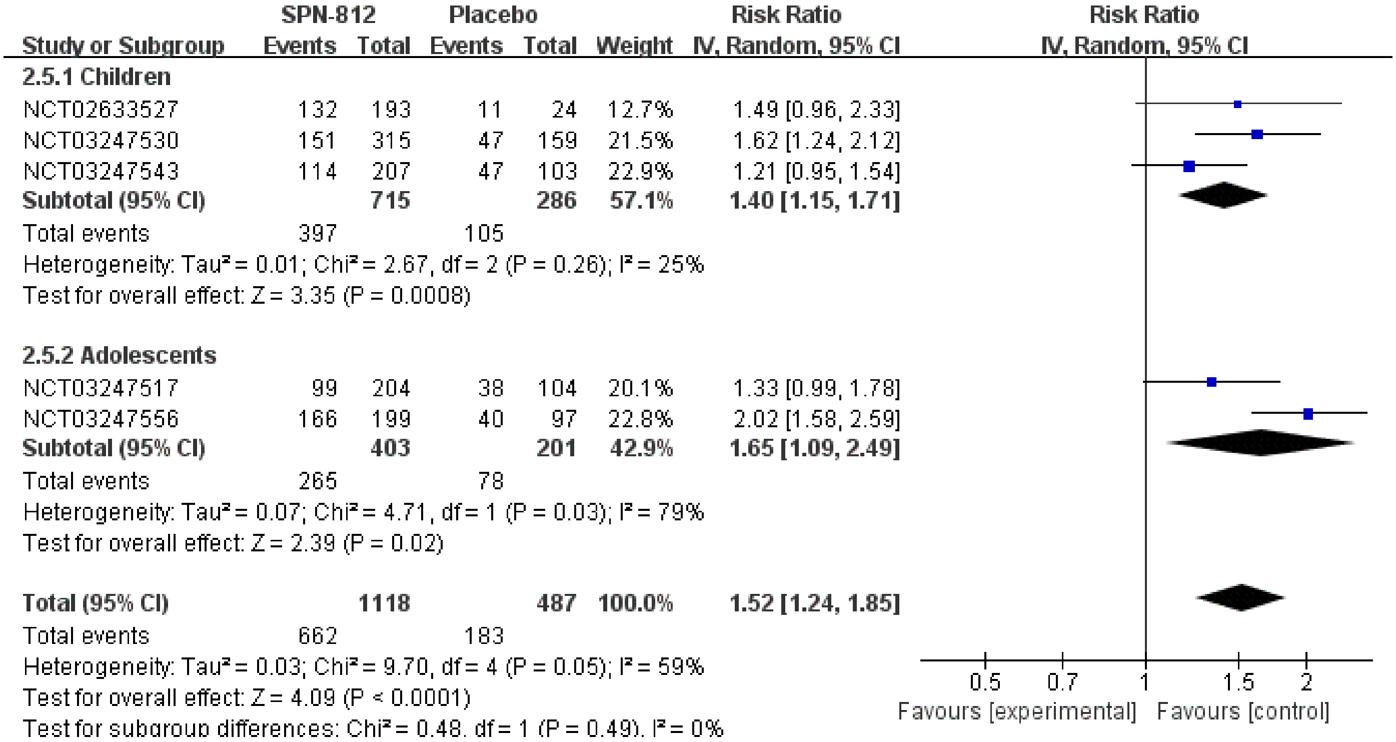

Figure S12 Subgroup analysis of safety: SAEs

Study or Subgroup	SPN-812 Events	SPN-812 Total	Placebo Events	Placebo Total	Weight	Risk Ratio IV, Random, 95% CI
**2.6.1 Children**						
NCT03247530	3	315	0	159	25.5%	3.54 [0.18, 68.20]
NCT03247543	3	207	0	103	25.6%	3.50 [0.18, 67.13]
**Subtotal (95% CI)**		**522**		**262**	**51.1%**	**3.52 [0.44, 28.47]**
Total events	6		0			
Heterogeneity: Tau² = 0.00; Chi² = 0.00, df = 1 (P = 1.00); I² = 0%						
Test for overall effect: Z = 1.18 (P = 0.24)						
**2.6.2 Adolescents**						
NCT03247517	2	99	0	104	24.4%	5.25 [0.26, 108.01]
NCT03247556	2	100	0	97	24.4%	4.85 [0.24, 99.77]
**Subtotal (95% CI)**		**199**		**201**	**48.9%**	**5.05 [0.59, 42.82]**
Total events	4		0			
Heterogeneity: Tau² = 0.00; Chi² = 0.00, df = 1 (P = 0.97); I² = 0%						
Test for overall effect: Z = 1.48 (P = 0.14)						
**Total (95% CI)**		**721**		**463**	**100.0%**	**4.20 [0.94, 18.71]**
Total events	10		0			
Heterogeneity: Tau² = 0.00; Chi² = 0.06, df = 3 (P = 1.00); I² = 0%						
Test for overall effect: Z = 1.88 (P = 0.06)						
Test for subgroup differences: Chi² = 0.06, df = 1 (P = 0.81), I² = 0%						

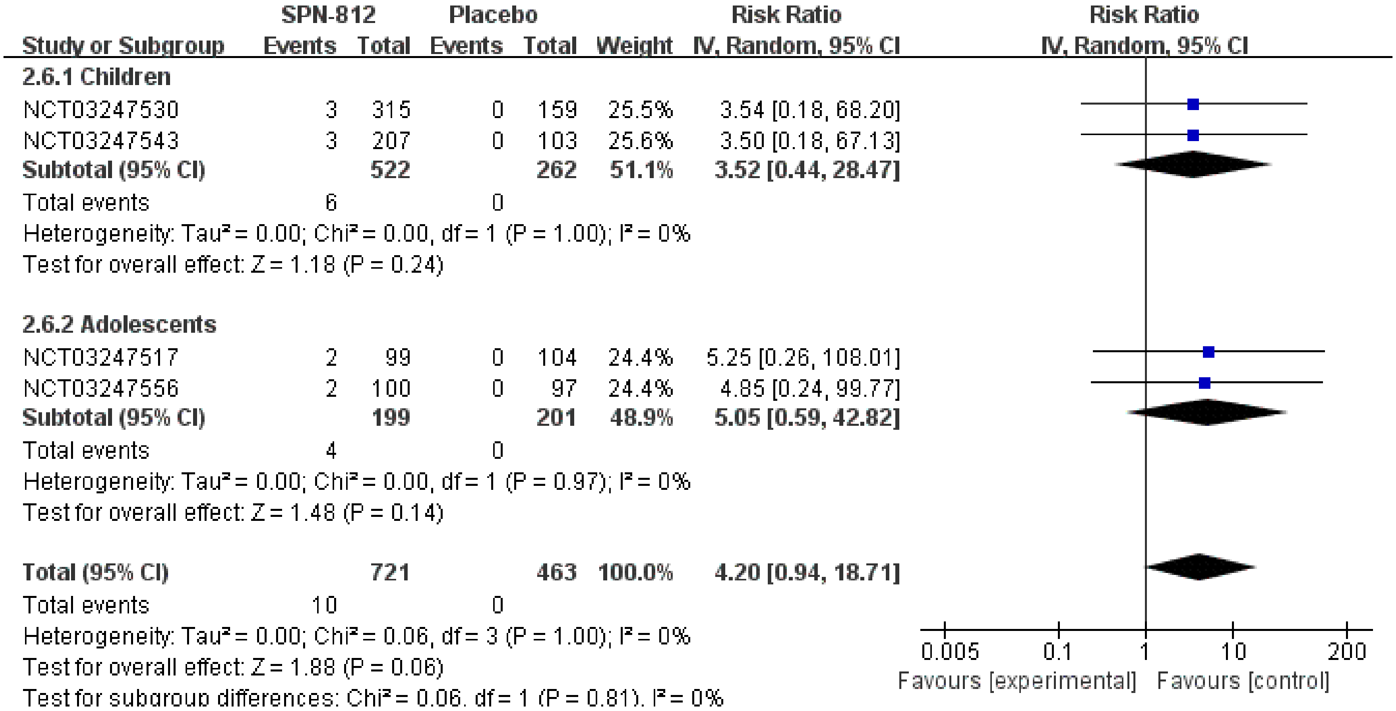

Supplement: Supplementary file 1 [file brainsci-13-01627-s001.zip › brainsci-2714605-supplementary.pdf]
